# DP5 without DFT: uncertainty-calibrated graph neural net accelerates structure confirmation *via* NMR

**DOI:** 10.1039/d5sc06988b

**Published:** 2026-03-05

**Authors:** Ruslan Kotlyarov, Alexander Howarth, Jonathan M. Goodman

**Affiliations:** a Yusuf Hamied Department of Chemistry Lensfield Road Cambridge CB2 1EW UK jmg11@cam.ac.uk

## Abstract

The evaluation and assignment of candidate structures to NMR spectra can be facilitated by the DP4 method, which assumes that one of the candidate structures is correct, and the DP5 method, which calculates the probability of a correct assignment for each candidate individually. Both of these methods require DFT calculations and thus a significant amount of computer resources. In this paper we present DP5q, a new version of DP5, which uses a graph convolutional neural network and quantile regression to replace the DFT-based algorithm. This dramatically increases the speed of the calculation at the cost of a modest decrease in accuracy. We demonstrate the efficacy of this rapid calculation both on a test set of thousands of molecules and also on cases selected for the difficulty of assigning the structure.

## Abstract 2

DP5q calculates the probability that a molecule is correctly assigned to an NMR spectrum. Extensive testing shows the results are comparable in accuracy to DFT-based DP5 and dramatically faster.

## Graphical abstract

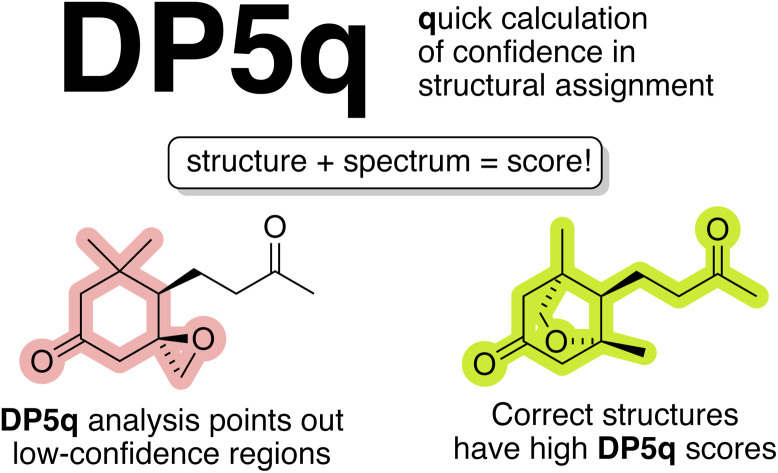

## 1 Introduction

Nuclear magnetic resonance spectroscopy is an important technique for characterisation of small molecules. It requires very little material for successful spectrum collection. The sample is easy to prepare, and the technique is non-destructive, *i.e.*, the product may be recovered intact due to the very low energy of electromagnetic radiation employed in the method.

Structure confirmation is relatively easy to accomplish when an experimental spectrum of a pure substance has already been recorded under the same conditions, such as temperature, solvent, and acidity. However, when a new compound is made, there are no reference spectra to compare.

The assignment of candidate structures to NMR spectra can be a challenging task, particularly when multiple structures have similar spectra. This has led to many examples of misassignment that have later been corrected.^1–23^ This issue is becoming more acute with increased automation, miniaturisation, and parallelisation of chemical synthesis. The amount of data generated within a single experimental campaign grows beyond individual chemists' capacity to process it. A fast, automated way to confirm a structure from a spectrum would address this growing challenge.

Our previous studies in the area of computer-aided structure elucidation led to CP3 (ref. 24) and DP4 (ref. 25) methods, which select the structure from the best-matching structure from the user-provided list, DP4-AI,^26–28^ which automates the process, and, most recently, DP5 (ref. 29), which determines the confidence with which a single structure may be assigned to a single spectrum. All of these methods require DFT analyses and the resources needed for these calculations are the rate determining steps for the process. In this work, we present a new version of DP5 analysis, DP5q, which obviates the need for DFT calculations while maintaining the accuracy to a high level. This dramatically improves the throughput for the DP5 process.

## 2 Neural net design

### 2.1 Learning explicit distribution parameters

Neural nets have already been used to help with structure elucidation.^30–33^ Several models can predict NMR spectra.^34,35^ IMPRESSION^34^ featurises molecular geometries using FCHL representations^36^ and uses kernel ridge regression to predict both the shift value and its variance. Graph-convolutional neural nets have been used to generate new learned features and predict molecular and atomic properties together with the associated uncertainties.^35,37^

Estimation of uncertainty for chemical problems is well-precedented and is used in several models that can predict NMR spectra.^34,35^ IMPRESSION,^34^ which was developed by Gerrard *et al.*, featurises molecular geometries using FCHL representations^36^ and uses kernel ridge regression to predict both the shift value and its variance. Work by Jonas and Kuhn,^35^ on the other hand, used a graph-convolutional neural net to generate new learned features and predicted shift and its uncertainty using two separate heads. Both approaches assume normal distributions for their predictions.

### 2.2 Quantile regression

**Fig. 1:**
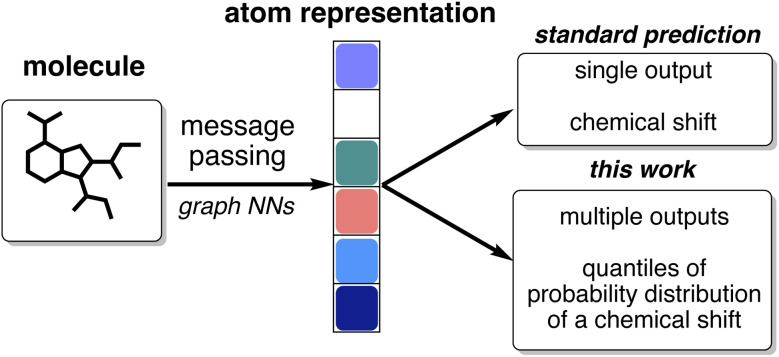
Quantile regression as an uncertainty estimation method is easy to incorporate into existing deep learning model architectures for chemical shift prediction.

In our model, we have used quantile regression for our uncertainty estimation. Instead of predicting explicit parameters of a probability distribution (PDF) as an output, it predicts the values of inverse cumulative distribution of a chemical shift for any specified set of quantiles (Fig. 1).

The approach is easy to integrate into the architecture of CASCADE,^38^ a graph neural network: instead of a single output, the final layer of the model can be reconfigured to produce multiple outputs corresponding to each specified quantile. For example, we can configure and train the model to predict the median, the first quartile, and the seventh decile of the probability distribution for a chemical shift. Realising that each additional quantile requires significantly fewer trainable parameters compared with the total number of the parameters within the model, we have evaluated chemical shift predictions for all percentiles from the 1st to the 99th, as the 0th and the 100th percentiles would simply correspond to minimum and maximum shifts observed in the training dataset. This allows us to balance effective interpolation of the cumulative distribution function for accurate analysis and the number of trainable parameters in the model.

### 2.3 Comparison with prior approaches

This work builds upon our prior efforts in uncertainty quantification for NMR shift predictions. Previously, we relied extensively on DFT calculations. For example, the DP4 approach^25,28^ uses errors between scaled DFT-predicted and unscaled experimental shifts and compares them to the previously benchmarked normal error distributions for correct structure assignments. These errors are converted into atomic probabilities, which are then multiplied together, and the sum of molecular probabilities for all candidate structures is normalised to unity. The structure with the highest DP4 score is selected as the most probable structure. This approach assumes that one of the proposed structures is correct and does not account for any structural features.

DP5 analysis^29^ goes further and constructs a dataset with tens of thousands of FCHL representations^36^ of atomic environments and their associated DFT errors. For an observed atomic environment, a probability distribution is constructed based on similar atomic environments to generate the probability of observing such an error. These atomic probabilities are then combined into the absolute molecular probability of correct structure assignment.

Our new neural net-based approach removes the need for DFT calculations altogether. Instead, the model constructs the probability distribution for chemical shifts directly. The speed of quantile regression-based DP5q analysis no longer depends on the size of the reference dataset, which allows us to arbitrarily scale up the size of the training data without increasing the computational cost.

## 3 Training

### 3.1 Loss function

The proposed loss function for the quantile regression is made of two parts:^39^ the prediction loss for all modelled quantiles and a regularisation term.

#### 3.1.1 Simultaneous multiple quantile loss calculation

The first part weights modified Huber loss 
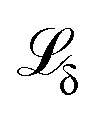
 (eqn (1)) for each quantile prediction *ŷ*_*τ*_ using quantiles specified at the point of model creation (eqn (2)). In the limit of a low *δ* parameter (here, set to 1 × 10^−4^ for good numerical convergence during training), modified Huber loss reduces to mean absolute error and our result for a single quantile *τ* becomes indistinguishable from pinball loss.1
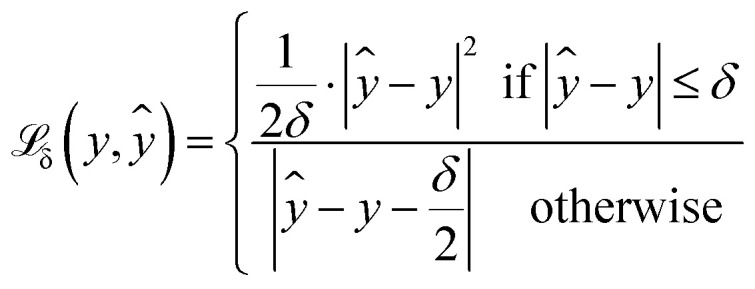
2
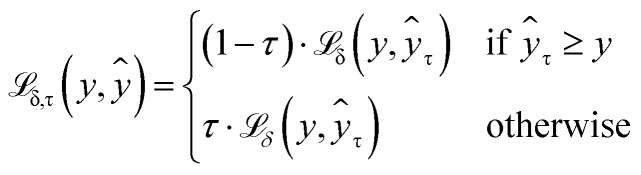


**Fig. 2:**
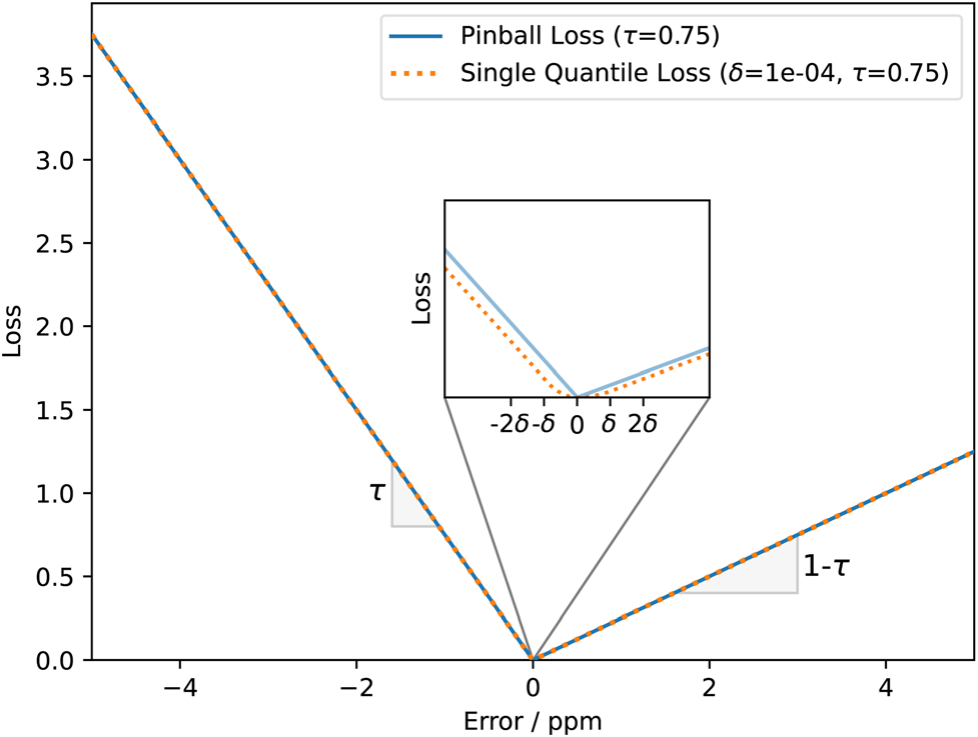
Example of a proposed loss function for the quantile *τ* = 0.75 (75th percentile of probability distribution). In the limit of large errors the function reduces to pinball loss for the same quantile. This modification is required to ensure well-defined gradients of the loss function throughout the entire predictive range.

An example of the proposed loss function for a single quantile *τ* is shown in Fig. 2. For all quantiles, the correct prediction has no associated loss. However, the parameter *τ* is used to weight the loss for over- and under-predictions. For example, if *τ* is set to 0.75, the loss for under-prediction is three times larger than the loss for over-prediction. Conversely, if *τ* is set to 0.5, the penalty for over- and under-prediction is equal and guides the model to predict the median of the distribution.3
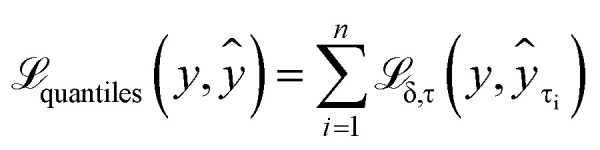


By minimising the sum of losses 
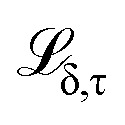
 for each quantile *τ* (eqn (3)), we hope that predictions for all quantiles will both accurately reflect the probability distribution and approach the true value *y*, thus preventing the model from overestimating its uncertainty. In this study, we have used *n* = 99 quantiles, corresponding to percentiles from 1 to 99, *i.e.*, *τ* = (0.01, 0.02, …, 0.99).

#### 3.1.2 Enforcing monotonously increasing predictions

The loss function presented in eqn (3) is sufficient to train the model to perform quantile regression. However, there is no guarantee that the predicted quantiles will make intuitive sense. For example, a 90th percentile prediction may still end up lower than an 80th percentile prediction, which is contrary to the properties of the cumulative distribution function, as it is expected to never decrease. Models that violate the non-decreasing property exhibit behaviour known as ‘quantile crossing’.

We can enforce the non-decreasing character by adding the second loss term 
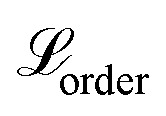
 (eqn (4)), which penalises higher quantiles having lower values, *i.e.*, preserves ranking order and monotonicity. With a larger number of prediction quantiles, it should be easier to maintain the desirable effects of the proposed loss function. Here, *ε* is a small positive number set to 1 × 10^−6^ to ensure a positive difference between two consecutive quantiles.4
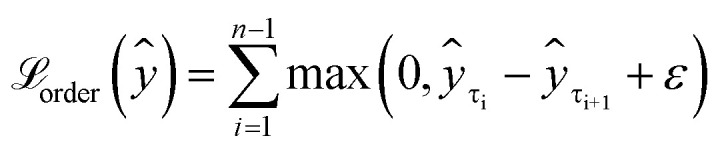


We, therefore, adapted the sum of the two components as the final loss function for the model (eqn (5)).5
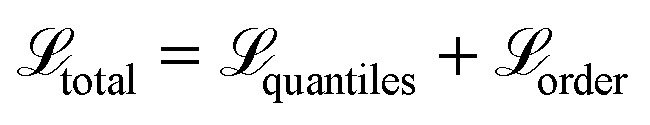


### 3.2 Dataset preparation

**Fig. 3:**
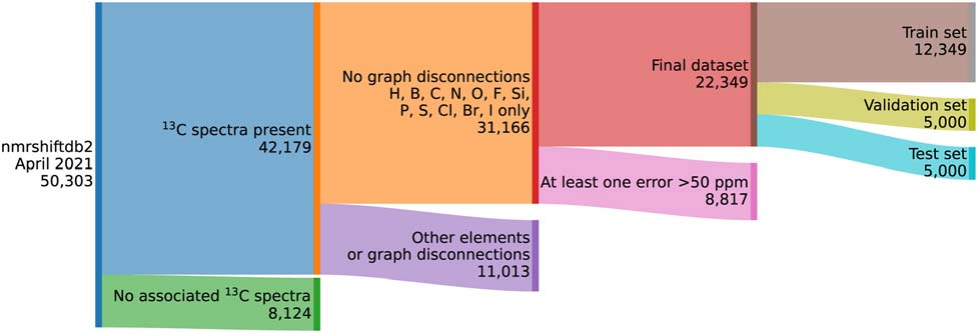
Overview of the dataset curation procedure.

The training dataset has been curated from NMRShiftDB^40^ data (Fig. 3). Since the goal is to analyse carbon NMR spectra, all entries without the carbon NMR spectrum were discarded. To aid training of the graph neural net, all molecules containing multiple fragments (*e.g.*, salts) were removed as well. Molecules containing atoms other than H, B, C, N, O, F, P, S, Cl, Br, and I were discarded, as rare atom types hinder effective training. Possible duplicates were treated as independent entries.

For each curated molecule, a 3D conformer was generated using ETKDG, as implemented in RDKit.^41^ The structures had their geometries optimised using the MMFF force field. Shielding tensors were calculated using DFT (mPW1PW91/6-311g(d)) in line with our previous studies^28^ without further geometry optimisation and then converted into chemical shifts using reference shielding tensor values for tetramethylsilane. After the calculations, any molecule with a large difference between predicted and observed shifts (50 ppm) was deemed to be an erroneous interpretation or assignment of the spectrum and discarded, leaving 22 349 records for 22 248 unique molecules and their associated experimental chemical shifts.

### 3.3 Training procedure

The model was then trained using the procedure described by Guan^38^ for 1200 epochs, an initial learning rate of 5 × 10^−4^, and reduction of the learning rate by 4% every 100 000 training steps using the Adam optimiser. Early stopping after 10 epochs without improvement in validation loss was used to prevent overfitting.

## 4 Transforming model uncertainty into structure confidence

**Fig. 4:**
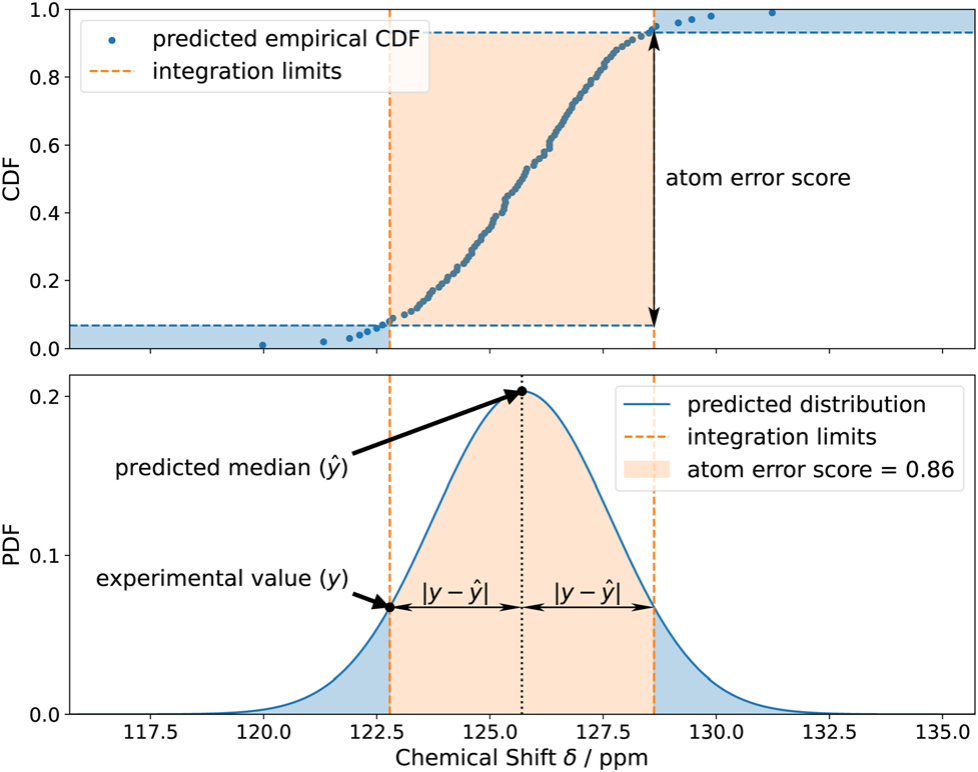
Calculation of the error score for the subsequent DP5 analysis using the experimental shift value and the predicted probability distribution.

Accurate uncertainty quantification is important for our use case. However, it is not pursued for its own sake, but rather as a step towards structure confirmation and to generate the probability distribution we will then integrate for an error score *P*_*i*_ as defined in eqn (6) (orange area in Fig. 4). Here, the integration limits are set between the predicted median minus absolute error *ŷ*−|*ŷ*−*y*| and predicted median plus mean absolute error *ŷ+*|*ŷ*−*y*|. The advantage of quantile regression in this case is that the model outputs the quantiles of the cumulative distribution directly without the need to integrate the probability distribution.6
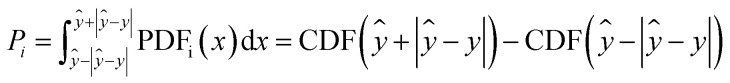


The DP5q score for atom *i* is defined in eqn (7) (blue area in Fig. 4) and the molecular DP5 score for *n* atoms would be their geometric average (eqn (8)). A high DP5q score therefore means that the observed shift is consistent with the chemical environment in the molecule.

7 DP5q_*i*_ = 1 − *P*_*i*_

8
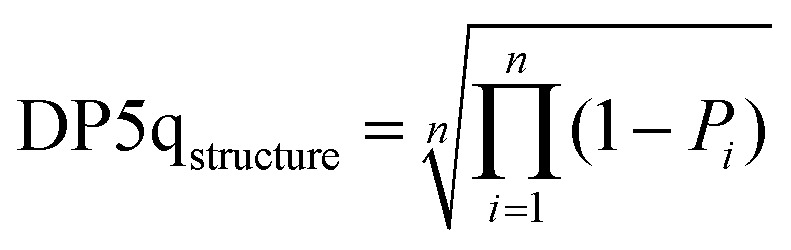


## 5 Testing

### 5.1 Impact of uncertainty quantification on shift prediction accuracy

**Fig. 5:**
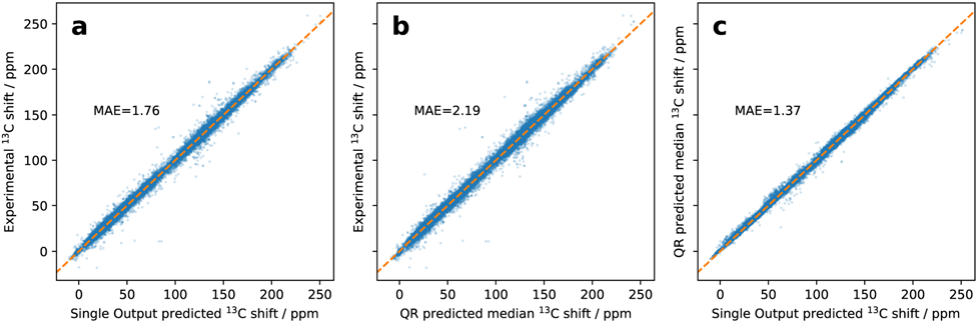
Impact of incorporation of the QR-based uncertainty estimation into NMR shift prediction model architecture on accuracy of NMRdb shift predictions. (a) Parity plot of single-output predictions against experimental shifts; (b) parity plot of medians of uncertainty-calibrated single-output predictions against experimental shifts; (c) parity plot of medians of uncertainty-calibrated predictions against single-output predictions.

To see if the model has been trained correctly, its median predictions were compared to experimental data (Fig. 5). The accuracy against experimental data deteriorated somewhat, with MAE increasing from 1.76 to 2.19 ppm. The increase in mean absolute error could mean an overfit to training data. However, the overfit must have been mitigated by the use of a validation set and early stopping. Another explanation would be a shift of the objective. Since the median is only one of 99 quantiles to be predicted, it now has a lower contribution to the loss function than in the single-output scenario, where mean absolute error was employed for a single prediction. Other quantiles are as important as the median in our setup, since we now need to accurately predict the probability distribution for chemical shifts.

### 5.2 DP5q is more sensitive than mean absolute error analysis

When deploying DP5q analysis in practice, we need to demonstrate that the scores we obtain carry practical meaning, *i.e.*, a high score means that the structure is likely to be correct, and a low score means that the structure is likely to be incorrect. We can readily generate the distribution of DP5q scores for the correctly assigned molecule, but how could we generate the scores for incorrect assignments? Some studies generate ‘incorrect’ structures artificially by altering the molecular structures already present in the dataset.^42,43^ However, we typically have no information about the experimental spectra of these structures and therefore cannot compute DP5q scores for them.

In combinatorial studies,^29^ we obtain ‘incorrect’ data without generating new molecules by taking advantage of existing databases. We notice that the database is a set of molecules with interpreted NMR spectra. Since there is a one-to-one match between a molecule and a spectrum and *vice versa*, all molecules but one in a data set will be an incorrect interpretation of a given spectrum. Therefore, using a dataset of size *N* provides us with *N* ‘correct’ spectrum–structure pairs and at most *N*^2^ − *N* ‘incorrect’ spectrum–structure pairs. For each pair, we compute mean absolute error and the DP5q score using eqn (8).

Within each group, we then assigned spectra to right and wrong structures and checked if the scores for correct structures are visibly different from the scores for incorrect structures. The utility of the DP5q score is to discriminate between right and wrong proposals.

We have taken the test set of 5000 molecules the NMR predicting model (Exp5k dataset from ref. 38) has not seen and assigned a spectrum to all of the appropriate structure-spectrum pairs using the algorithm developed by Lewis *et al.*^44^ Here, we have obtained 5000 correct assignments and 5614 incorrect assignments where the incorrect proposal had the same molecular formula.

**Fig. 6:**
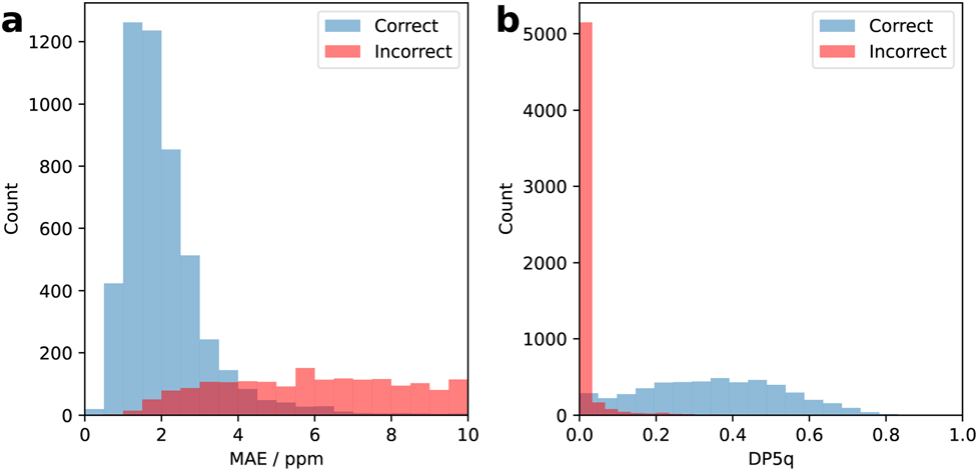
(a) Distribution of mean absolute errors for 5000 correct structure assignments (blue) and 5614 incorrect structure assignments (red) generated by assigning every spectrum to molecules with the same molecular formula. (b) Distribution of DP5q scores for the same correct (blue) and incorrect (red) structure assignments. The DP5q score converts mean absolute error into a metric of confidence in structural interpretation.

Their distributions of mean absolute errors and DP5q scores are shown in Fig. 6. While the distributions of mean absolute errors for correct and incorrect proposals overlap significantly, the distributions of DP5q scores are much more distinct, with scores for incorrect proposals concentrated around zero. This demonstrates that the DP5q score is a useful way to select correct proposals.

**Fig. 7:**
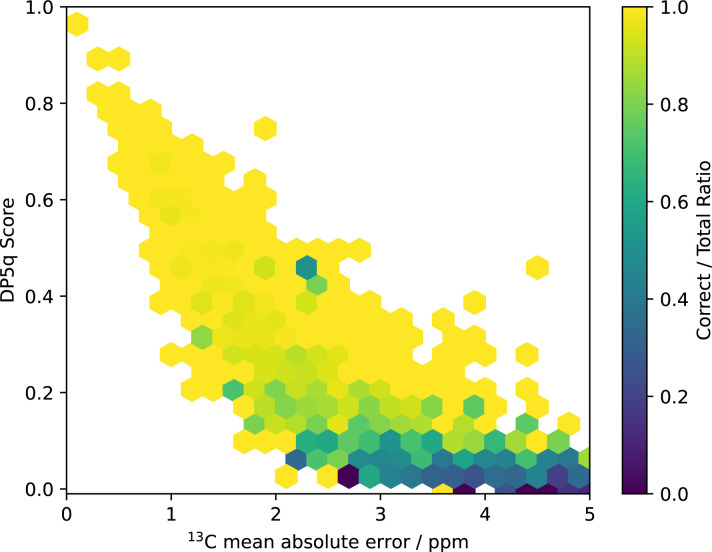
Ratio of correct interpretations to the total number of interpretations. A high DP5q score and low mean absolute error indicate a high likelihood of correct structure assignment. The DP5q score is a better predictor of correct structure assignment than mean absolute error alone.

Two summary metrics can be combined together to assess confidence in the structure interpretation (Fig. 7). We group the neighbouring data points into bins and calculate the ratio of correct interpretations to the total number of interpretations within each bin. This ratio may be interpreted as the probability of a spectrum being interpreted correctly given the combination of mean absolute error and DP5 score.

Here, we clearly observe a steep decline of the DP5q score with increasing mean absolute error. This is reasonable to expect as correct interpretations of the spectra must match the data closely. We then see that beyond a mean absolute error of 4 ppm, the DP5 score almost does not vary with increasing error. This is expected, as the structures with high errors are near certain to be incorrect.

The graph also highlights a clear separation between correct and incorrect proposals. Correct proposals tend to cluster at higher DP5q scores and lower mean absolute errors, while incorrect proposals are more dispersed and skewed towards lower DP5 scores. This separation demonstrates the robustness of the DP5 metric in distinguishing correct structures from incorrect ones.

The combination of two metrics also helps us isolate high-confidence proposals with greater care. For example, proposals with low DP5 scores combined with low mean absolute errors are considered more likely than proposals with the same low DP5 scores combined with high mean absolute errors.

Additionally, the steep decline in DP5 scores for small increases in mean absolute error indicates that the method is highly sensitive to small deviations in predicted spectra. This sensitivity is advantageous for identifying subtle differences between closely related structures, making the DP5 approach particularly useful for challenging cases of structure elucidation.

### 5.3 Challenging structural revision case studies

**Fig. 8:**
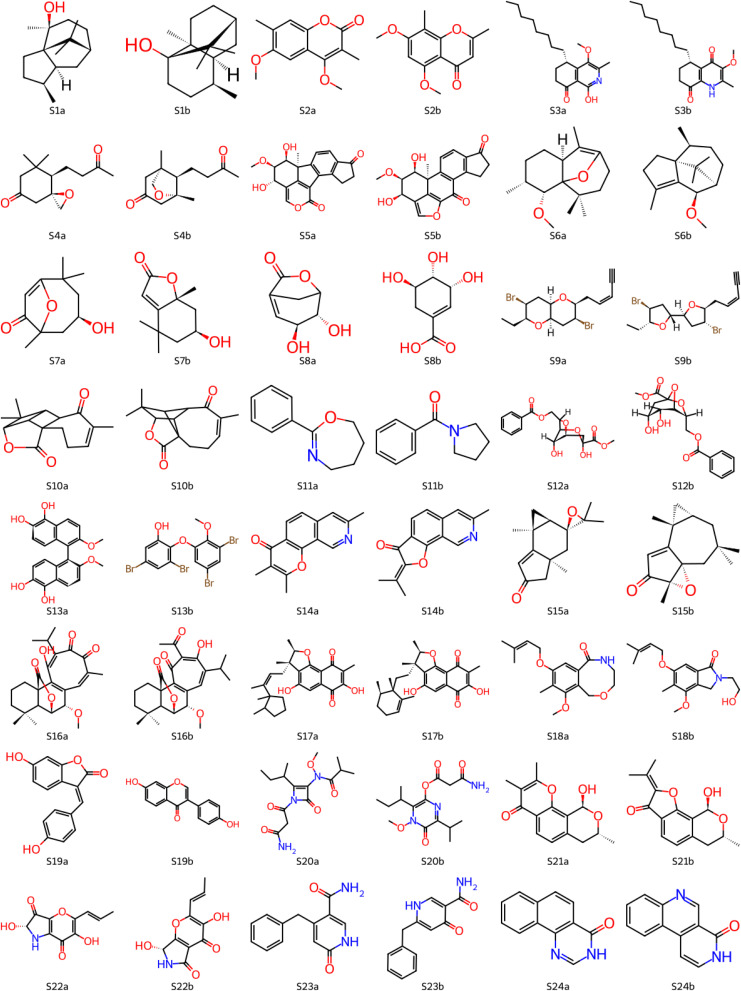
Examples of molecules which had their initial published structures reassigned. Here, letter ‘a’ in the name (*e.g.*, S1a) denotes an initial proposal and letter ‘b’ (*e.g.*, S1b) denotes a reassigned structure.

In these studies, we have demonstrated the effectiveness of the new DP5q approach as a summary metric. These offer a bird’s-eye, abstract view of its performance. To gain the confidence of laboratory-based chemists, we consider concrete examples, taken from the structure revision studies (see Fig. 8). Molecules S1^2^, S2^3^, S3^4^, S4^5^, S5^6^, S6–S8^7^, S9^8^, S10^9^, S11^10^, S12^11^, and S13^12^ were used in our previous DFT-based DP5 study.^29^ Molecules S14^13^, S15^14^, S16^15^, S17^16^, S18^17^, S19^18^, S20^19^, S21^20^, S22^21^, S23^22^, and S24^23^ are new.

Here, a structure for a molecule was proposed based on multiple methods, including several NMR experiments, mass, UV-vis spectroscopy, and infrared spectroscopy. However, subsequent attempts to re-create the proposed structure did not succeed, with recorded spectra not matching that of the authentic sample. New structures are then proposed and confirmed by *de novo* chemical synthesis or by comparison with spectra of known natural products.

**Fig. 9:**
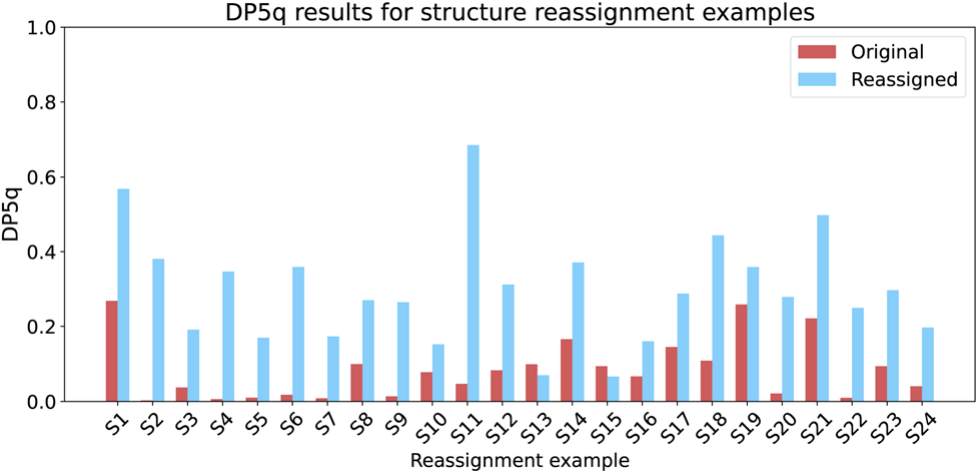
DFT-free DP5q for structure reassignment case studies. In 22 cases out of 24, the correct structure had a higher score than the initial proposal.

We took 24 case studies and conducted DP5q analysis using single conformers, optimised at the force-field level. For 22 cases, the score for the correct proposal was higher than the score for the incorrect proposals (Fig. 9). This result is comparable to that of 23 examples where the mean absolute error of a revised structure is lower than the mean absolute error of the original structure. We note that this is a very challenging test set: someone has already analysed the spectra in detail and published a conclusion that was later found to be incorrect. For more common cases, by distinguishing automatically between large numbers of diverse structures the results are likely to be even more reliable.

**Fig. 10:**
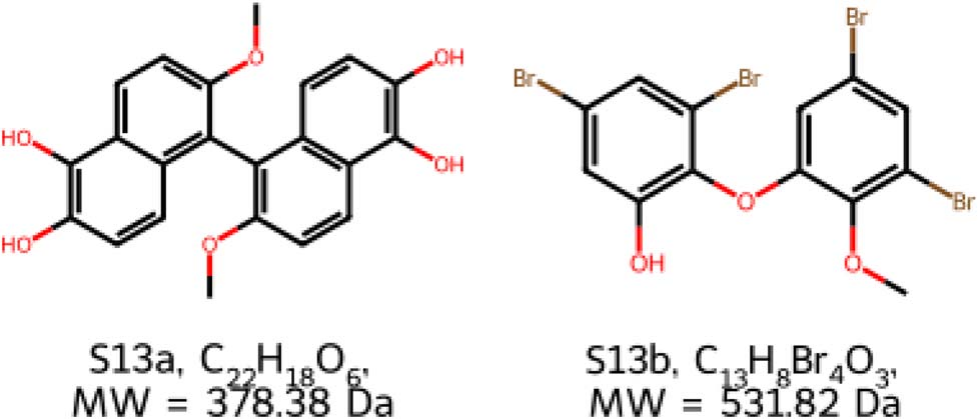
For S13, the initial and revised proposals have drastically different molecular weights and formulae.

This is a key result: we achieve the DFT performance at the neural network cost. What previously took CPU months is now achievable in seconds. The only example that the model could not tackle was S13. Therefore, it merits a closer look (Fig. 10).

Here, we see that the original proposed structure was a symmetric binaphthyl, while the revised structure lacks any symmetry elements. Furthermore, the structures have different numbers of distinct NMR environments, 11 and 13 respectively. Both proposals, however, have four non-labile proton environments and four non-quaternary carbon environments. If the spectra have a low signal-to-noise ratio, the other, less intense, signals may be indistinguishable from the baseline. This shows that our approach depends on correct interpretation of the NMR spectra: if signals are lost into noise, an accurate analysis may not be possible. The molecular masses of the two proposals are quite different as well. This highlights the importance of using several structural determination techniques at once, so that spurious proposals based on a single modality are rejected by an orthogonal method and good proposals are corroborated. Addressing this issue is a topic of on-going research.

**Fig. 11:**
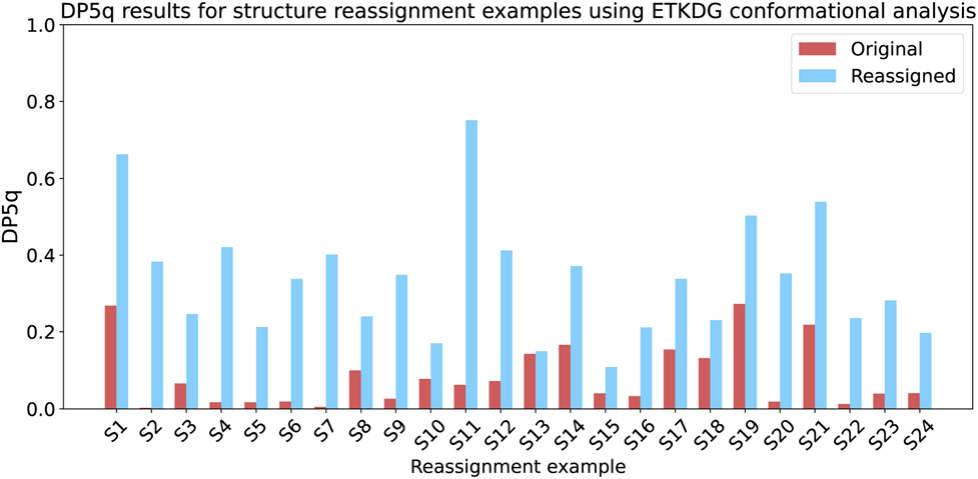
DP5q analysis of the structure revision studies, using ETKDG-based conformational search. In 24 cases out of 24, the correct structure had a higher score than the initial proposal. A direct comparison of mean absolute errors leads to the correct structure in 23 cases out of 24.

Using the ETKDGv3 algorithm within RDKit as a quick conformational search tool improves the accuracy of our analysis, with higher scores predicted for the correct compound in all 24 cases (Fig. 11). On the other hand, a simple comparison of mean absolute errors returned the correct result 23 times out of 24. This highlights the importance of the representative conformer ensemble in accurate prediction of NMR shifts, even when DFT spectrum calculation has been eliminated from the workflow.

**Fig. 12:**
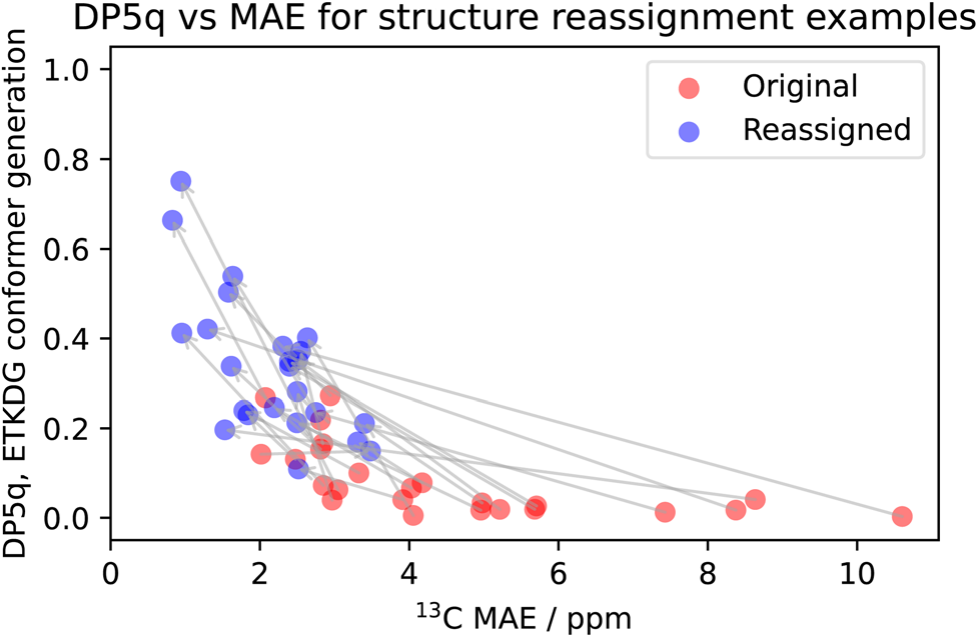
DP5q and MAE comparison of the structure revision studies, using ETKDG-based conformational search. For all cases, a higher DP5 score also means a lower mean absolute error. MAE is calculated by comparing the median of the predicted shift distribution to the experimental shifts.

The combination of DP5q scores and mean absolute errors (MAE) of the carbon spectra (Fig. 12) shows that they are linked to some extent, as higher scores tend to correspond to lower errors. The distribution of correct and incorrect proposals resembles the one observed in the combinatorial studies (Fig. 7). There is a small overlap between DP5q scores of correct and incorrect proposals, but the correct proposals still tend to have a lower error and higher DP5q score. This is a good result and shows that the DP5q score is an effective method even for structurally complex molecules, such as natural products.

### 5.4 Relative stereochemistry elucidation

**Fig. 13:**
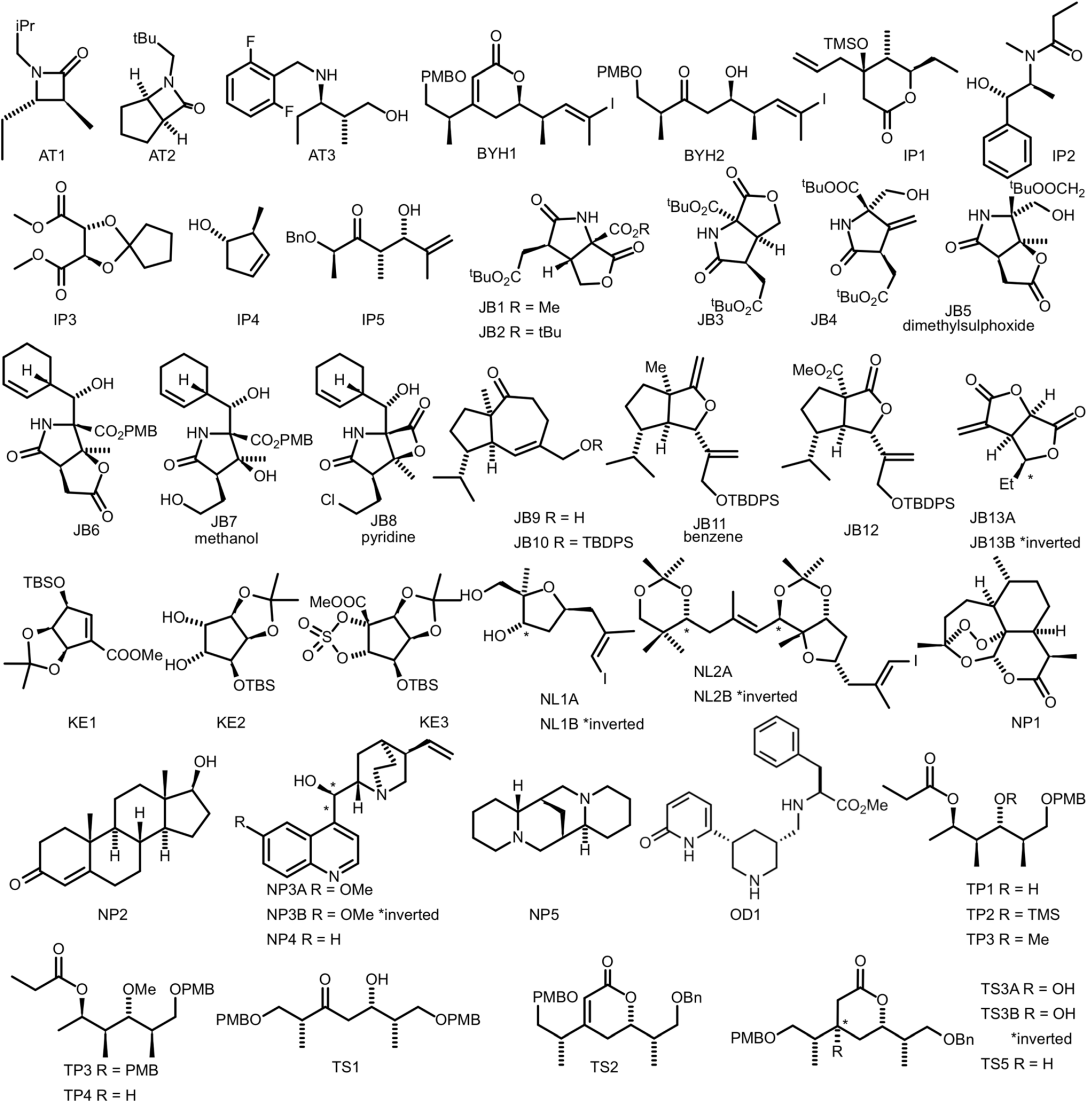
Molecules used in the DP4-AI test set. AT3, TS3A, TS4 and NL1A had no corresponding carbon spectra and were, therefore, excluded from analysis.

Another worthwhile and very challenging test of the method would be to apply it to an established test set used to determine performance of DP4-AI.^28^ It consists of 42 molecules with their interpreted carbon spectra (Fig. 13). The dataset is made of natural products and synthetic intermediates to represent a broad range of chemical space. The differences between the diastereomers are rather small, which makes it a robust test of DP5 analysis. A good result for this challenging dataset would demonstrate the power of the method. We have already demonstrated the power of DP5q for less challenging datasets (Fig. 7).

We consider three scenarios with varying degrees of sophistication. In the first scenario, we use a single conformer, optimised at the force-field level. This is the fastest way to run DP5 analysis. In the second scenario, we use multiple conformers generated using low-mode conformational search, with geometries optimised at the DFT-level and energies recalculated using single-point DFT calculations. This is the most sophisticated way to run DP5q analysis, and its previous use for stereochemistry determination^28^ has shown it to be the most accurate. In the third scenario, we use multiple conformers generated using the ETKDGv3 algorithm^41^ as provided with RDKit, further optimised at the force-field level of theory.

To visualise the resulting DP5q scores, we divide them by the number of possible diastereomers. For example, if the molecule has 8 diastereomers, the plotted DP5q scores will be divided by 8. This helps us both indicate the relative magnitudes and absolute values for DP5 probabilities.

#### 5.4.1 Single conformer, force-field

**Fig. 14:**
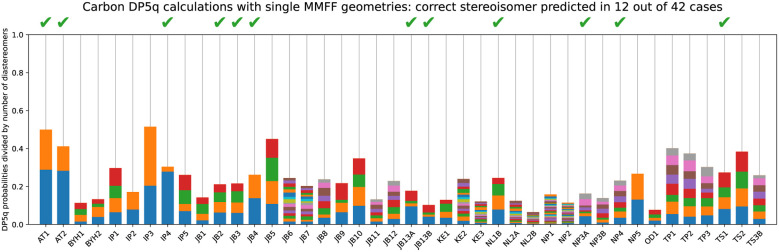
DP5q analysis of the DP4-AI test set using a single conformer, optimised at the force-field level.

The fastest way to run DP5q analysis is to dispense with all expensive steps, such as conformational search and DFT geometry optimisation and DFT single-point energy calculations. This analysis gets 12 examples correct (Fig. 14). While getting at least this many structures correctly is 80% likely not to be a coincidence, it is not sufficiently robust to be used in practice. Nevertheless, it is still better than suggesting stereochemistry at random, even for the dataset this complicated.

#### 5.4.2 Multiple conformers, DFT

We compare the minimalistic approach described in subsection 5.4.1 with the standard protocol for DFT calculations of NMR spectra, which entailed low-mode conformational search, geometry optimisation of the conformer ensemble, and re-ranking of conformers based on DFT single-point energy calculations. Here, however, we dispense with the estimation of isotropic shielding and use our neural net to calculate the chemical shifts together with predicted uncertainties.

**Fig. 15:**
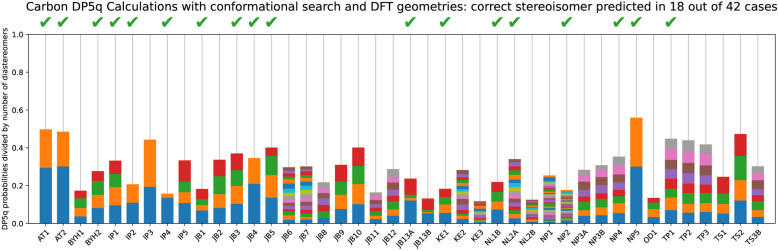
DP5q analysis of the DP4-AI test set using multiple conformers, optimised at the DFT level.

Here, stereochemistry was determined correctly for 18 examples out of 42 (Fig. 15), with a 0.13% chance of obtaining a result this good at random. This is an encouraging improvement, which is consistent with the trend where more sophisticated treatment improves the overall accuracy.

#### 5.4.3 Multiple conformers, ETKDG

It is useful to note that our model has been trained on force field geometries, and it might perform even better with the appropriate input. To test our hypothesis, we have used the ETKDGv3 algorithm^41^ to generate conformers and optimised them further within the MMFF94-s force field. This method produces time-averaged geometries, which is an appropriate assumption for the NMR timescale.

**Fig. 16:**
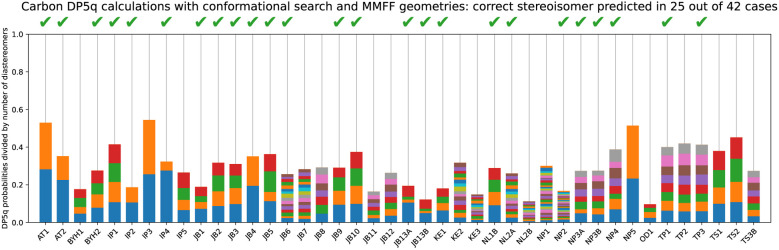
DP5q analysis of the DP4-AI test set using multiple conformers, generated using the ETKDG algorithm.

This approach may take a few minutes instead of a fraction of a second and helps us determine correct stereochemistry in 25 out of 42 cases, based on 1D^13^C NMR data only, using no ^1^H NMR or multidimensional spectroscopy (Fig. 16). This result has a 2.9 × 10^−6^% probability of occurring by chance. The method meets and exceeds purpose-developed DP4 analysis at a similar level of theory,^29^ with 19 out of 42 cases identified correctly, the DFT-based DP5 approach, where the correct diastereomer has been found in 16 cases out of 42,^29^ and the CASCADE mean absolute error comparison, where the lowest-error diastereomer was the correct one 21 times out of 42. Therefore, we recommend this for the comparison of stereoisomers and other very similar structures.

## 6 Conclusions

A new approach has been developed for estimating the confidence of interpretation of an NMR spectrum. It is fully integrated into an existing DP5 suite, accelerating structural elucidation by several orders of magnitude by eliminating costly DFT calculations. Our approach enhances the DP5 methodology by incorporating a 3D neural network capable of predicting NMR chemical shifts along with their uncertainties. This advancement allows for rapid and accurate assessment of molecular structures based on NMR data.

DP5q analysis performs admirably on the very complex structural revision case studies. Inclusion of quick conformational search results in scoring the correct proposal higher in 24 cases out of 24. DP5q analysis is also helpful for determining stereochemistry of a diverse range of molecules, with the correct diastereomer selected in 25 cases out of 42, which exceeds performance of DP4 analysis (15 examples correct) using a similar level of theory.^28^ Such performance makes DFT-free DP5q ideal for high-throughput NMR data curation. To aid such workflows, we recommend using ETKDGv3 conformer generation for best results and selecting a threshold of 0.2 for the DP5q score to reliably discard incorrect structure proposals.

This work sets a strong foundation for incorporating more sophisticated spectroscopy methods, including *J*-value analysis, 2D NMR spectra interpretation, and processing the mixtures. In addition to that, DP5q can be used as a scoring function for the plethora of molecular optimisation methods available, potentially enabling automated structure revision.

## Author contributions

Prof J. M. Goodman conceptualised and supervised the project. R. Kotlyarov implemented the machine learning model and evaluated its performance. A. Howarth curated the datasets for model training and evaluation, ran the DFT calculations, and performed preliminary research.

## Conflicts of interest

There are no conflicts to declare.

## Supplementary Material

SC-017-D5SC06988B-s001

## Data Availability

The code for DP5 structure confirmation software can be found at https://github.com/ruslankotl/DP5. The NMRdb dataset used in this study is available at https://github.com/ruslankotl/DP5. Supplementary information (SI): data on the acceleration of DP5q analysis over DP5, a TMAP plot showing how predicted uncertainty varies with the chemical environment, and detailed outputs of DP5 analyses. See DOI: https://doi.org/10.1039/d5sc06988b.
