# Supplementary material for: DP5 without DFT: uncertainty-calibrated graph neural net accelerates structure confirmation via NMR
Source: Chem Sci. 2026 Mar 5;17(18):9138–47. doi: 10.1039/d5sc06988b (PMC12997274; doi:10.1039/d5sc06988b)
Supplement: SC-017-D5SC06988B-s001 [file SC-017-D5SC06988B-s001.pdf]

Supplementary Information (SI) for Chemical Science.
This journal is © The Royal Society of Chemistry 2026

Supplementary Information

to accompany

DP5 without DFT: Uncertainty-calibrated graph neural net accelerates structure confirmation via NMR

Ruslan Kotlyarov, Alexander Howarth, and Jonathan M. Goodman

A Acceleration of DP5 Calculations using Graph Neural Networks

For 48 molecules featured in Section 5.3, we compared the time taken to calculate DP5 probabilities using the original DFT-based method and the new GNN-based method. All calculations were performed on 11th Gen Intel (R) Core (TM) i7-11700 @ 2.50GHz as CPU. The results are presented in the table below.

Stage	DP5 CPU time	DP5q CPU time
Conformational Search	16 hours	25 minutes
DFT Geometry Optimisation	242 days	N/A
DFT Single Point Energy	208 days	N/A
DFT NMR Shift Prediction	33 days	N/A
DP5 Analysis	1 hour	10 minutes
Total Time	493 days	35 minutes

This corresponds to a speed up by a factor of about 20 000 for the relatively demanding ETKDG conformation search which is required only for flexible and challenging molecules.

B Predicted Uncertainty versus Atomic Representation

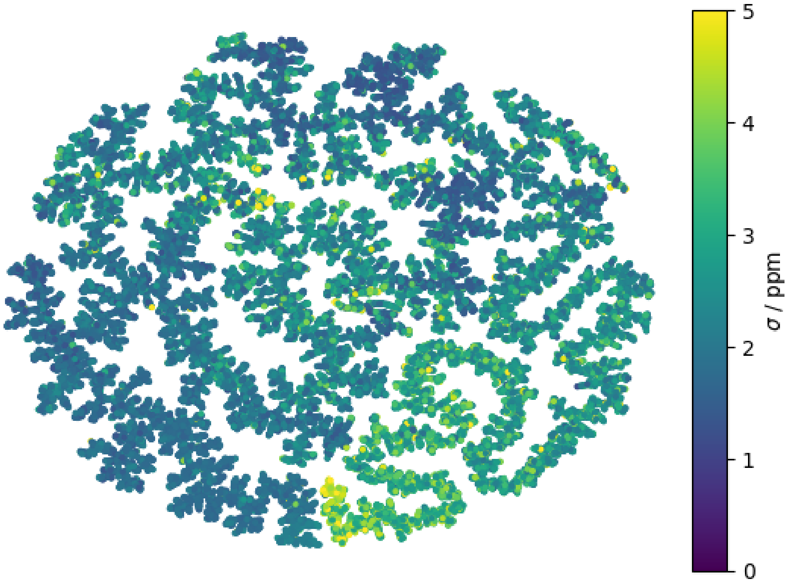

Figure 1: TMAP visualisation of atomic environments present in the test set. Each point represents a single atomic environment from the test set, coloured by the predicted uncertainty (in ppm) of that environment's NMR shift prediction. Environments with similar uncertaintites appear to be grouped together. Note that the method does not preserve global distances, but similar points are grouped together.

C DP5-Guided Determination of Stereochemistry

DP5 probabilities were calculated for all possible diastereomers of the 42 molecules in the DP4-AI dataset. In this table, the isomer number 1 is the correct structure. The highest DP5 probability calculated for each molecule is highlighted in bold.

For **Single Conformer** method, the 3D geometry of each diastereomer was generated using ETKDGv3 method and subsequently optimized using MMFF94s force field as implemented in RDKit.

For **Full DFT** method, the conformational ensemble was generated in Macromodel using low-mode search. The molecular geometries were further optimized at the DFT level of theory, this was performed using the B3LYP functional with the 6-31G(d) basis set. Finally, single-point energies were separately calculated using M06-2X functional and def2-TZVP basis set. NMR shielding constants were found using the GIAO method. The functional mPW1PW91 was chosen with the 6-311G(d) basis set for NMR shift prediction.

For **ETKDG**, the 3D geometries of all stereoisomers were generated using ETKDGv3 method and subsequently optimized using MMFF94s force field as implemented in RDKit.

Name	Molecule	Stereoisomer	Single Conformer	Full DFT	ETKDG
AT1	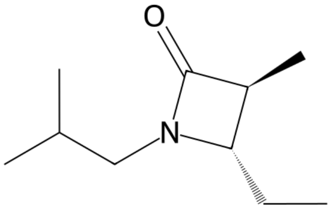	1	**0.5767**	**0.5889**	**0.5649**
		2	0.4230	0.4058	0.4938
AT2	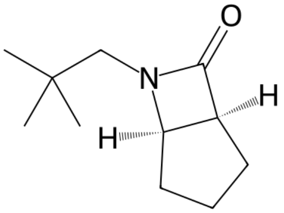	1	**0.5664**	**0.6028**	**0.4515**
		2	0.2565	0.3674	0.2522
BYH1	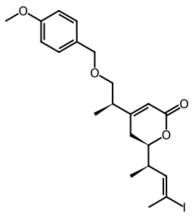	1	0.0616	0.1482	0.1874
		2	**0.1406**	0.1775	0.1401
		3	0.1219	**0.2062**	**0.1945**
		4	0.1303	0.1615	0.1857
BYH2	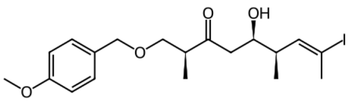	1	0.1586	**0.3264**	**0.3129**
		2	**0.2111**	0.2769	0.2838
		3	0.0683	0.2969	0.2348
		4	0.0940	0.2072	0.2725
IP1	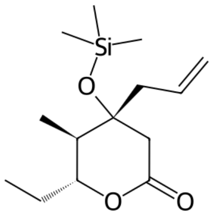	1	0.2578	**0.3847**	**0.4296**
		2	0.2985	0.3843	0.4295
		3	0.2594	0.2794	0.3996
		4	**0.3740**	0.2813	0.4003

continued on next page

continued from previous page

Name	Molecule	Stereoisomer	Single Conformer	Full DFT	ETKDG
IP2	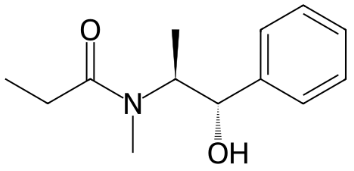	1	0.1570	**0.2187**	**0.2117**
		2	**0.1855**	0.1952	0.1619
IP3	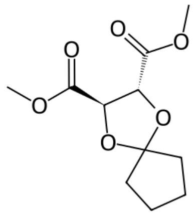	1	0.4089	0.3873	0.5117
		2	**0.6208**	**0.4988**	**0.5767**
IP4	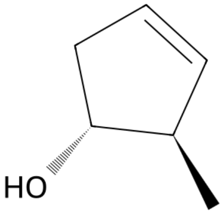	1	**0.5574**	**0.2715**	**0.5520**
		2	0.0514	0.0440	0.0947
IP5	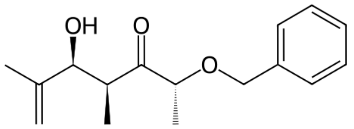	1	0.2805	0.4294	0.2621
		2	0.1512	0.2302	0.2184
		3	0.2897	0.2225	0.2502
		4	**0.3227**	**0.4520**	**0.3316**
JB1	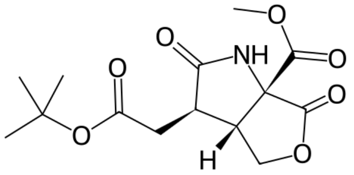	1	0.0875	**0.2717**	**0.2895**
		2	0.1354	0.1163	0.1440
		3	**0.2083**	0.1243	0.1256
		4	0.1395	0.2160	0.1970
JB2	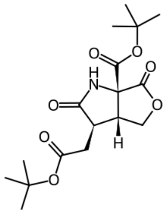	1	**0.2519**	0.3270	**0.3494**
		2	0.2199	0.2921	0.3117
		3	0.2052	**0.3797**	0.3361
		4	0.1700	0.3493	0.2713
JB3	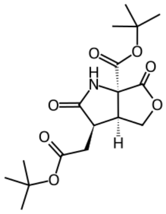	1	**0.2429**	**0.4151**	**0.3908**
		2	0.2208	0.3766	0.3400
		3	0.2373	0.3380	0.2656
		4	0.1641	0.3515	0.2452

continued on next page

continued from previous page

Name	Molecule	Stereoisomer	Single Conformer	Full DFT	ETKDG
JB4	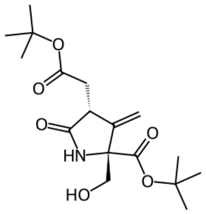	1	**0.2767**	**0.4186**	**0.3889**
		2	0.2469	0.2724	0.3133
JB5	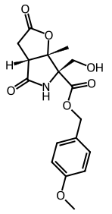	1	0.4340	**0.5449**	**0.4515**
		2	0.4801	0.4799	0.1958
		3	**0.4926**	0.4028	0.4372
		4	0.3952	0.1786	0.3676
JB6	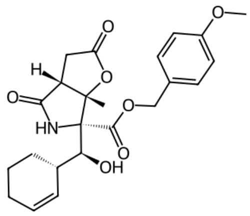	1	0.2482	0.3009	**0.3727**
		2	0.2475	0.3754	0.1542
		3	0.2394	0.3487	0.3128
		4	0.2036	0.1821	0.2476
		5	0.2401	0.2728	0.2932
		6	0.3022	0.2745	0.1818
		7	0.2459	0.3268	0.2748
		8	0.1037	0.2145	0.2639
		9	0.2973	0.2938	0.3478
		10	0.1954	0.3552	0.1824
		11	**0.3712**	**0.4350**	0.2152
		12	0.2669	0.3476	0.2995
		13	0.2109	0.2232	0.2949
		14	0.2895	0.2816	0.2580
		15	0.2509	0.2425	0.1634
		16	0.2078	0.2823	0.2411
JB7	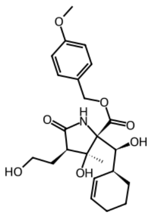	1	0.2462	0.3297	0.3005
		2	0.1436	0.2147	0.2673
		3	0.2940	0.2795	0.2891
		4	0.1721	0.3272	0.3103
		5	0.2464	0.3528	0.3129
		6	0.2190	0.3263	0.2652
		7	**0.3105**	0.3028	**0.3240**
		8	0.1957	0.3481	0.2886
		9	0.1247	**0.3748**	0.2908
		10	0.2006	0.3423	0.3122
		11	0.2057	0.3508	0.2605
		12	0.1448	0.3083	0.2282
		13	0.1598	0.2327	0.2325
		14	0.2253	0.3164	0.2552
		15	0.1673	0.1612	0.3199
		16	0.1861	0.2603	0.2674

continued on next page

continued from previous page

Name	Molecule	Stereoisomer	Single Conformer	Full DFT	ETKDG
JB8	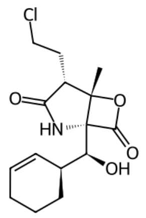	1	0.2841	0.2201	0.3735
		2	0.1354	0.0012	0.0013
		3	0.2668	**0.2894**	0.3719
		4	0.2946	0.2755	0.3481
		5	0.2292	0.2789	0.2697
		6	**0.3340**	0.2159	0.2963
		7	0.2164	0.2250	**0.3750**
		8	0.1434	0.2320	0.3020
JB9	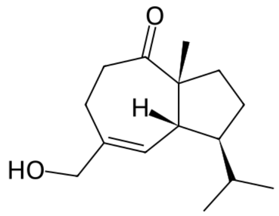	1	0.2564	0.3063	**0.3761**
		2	0.1997	0.3002	0.2969
		3	0.0640	0.2758	0.2854
		4	**0.3503**	**0.3566**	0.2066
JB10	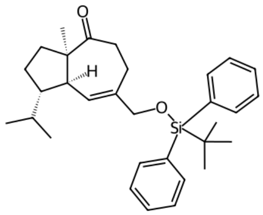	1	0.3940	0.4062	**0.3944**
		2	**0.3966**	**0.4259**	0.3835
		3	0.2631	0.3827	0.3712
		4	0.3386	0.3916	0.3506
JB11	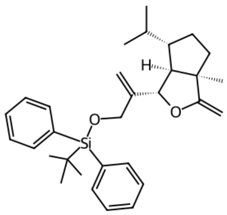	1	0.1233	0.1813	0.1804
		2	0.1381	0.1401	0.1387
		3	0.1225	**0.1912**	**0.1876**
		4	0.1254	0.1543	0.1614
		5	0.1467	0.1502	0.1617
		6	**0.1735**	0.1786	0.1624
		7	0.1217	0.1733	0.1633
		8	0.1044	0.1439	0.1578
JB12	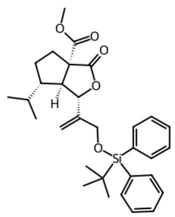	1	0.2332	0.3271	0.2866
		2	0.2179	0.2637	0.2792
		3	0.2795	**0.3354**	0.2718
		4	**0.3048**	0.2450	0.2465
		5	0.1734	0.2651	0.2284
		6	0.1736	0.2955	**0.2956**
		7	0.2543	0.2392	0.2419
		8	0.1941	0.3254	0.2561
JB13A	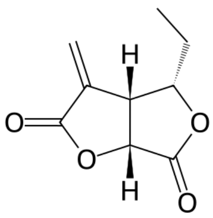	1	**0.3820**	**0.4857**	**0.4227**
		2	0.0638	0.0533	0.0638
		3	0.0516	0.0668	0.0598
		4	0.2130	0.3423	0.2322

continued on next page

continued from previous page

Name	Molecule	Stereoisomer	Single Conformer	Full DFT	ETKDG
JB13B	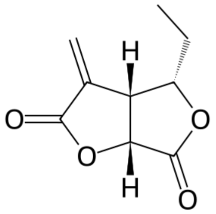	1	**0.1645**	0.2128	**0.1995**
		2	0.0422	0.0282	0.0422
		3	0.0461	0.0464	0.0505
		4	0.1577	**0.2408**	0.1958
KE1	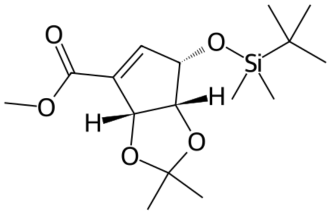	1	0.1417	**0.2217**	**0.2546**
		2	0.1250	0.1782	0.1570
		3	**0.1711**	0.1583	0.1357
		4	0.0806	0.1750	0.1753
KE2	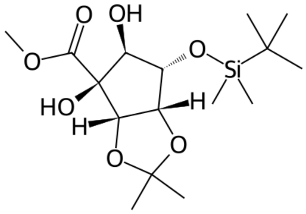	1	0.3106	0.3724	0.4133
		2	**0.3605**	0.2799	0.4029
		3	0.2503	0.1916	0.2454
		4	0.2176	0.2992	**0.4269**
		5	0.1913	0.4618	0.3745
		6	0.2575	**0.4670**	0.3478
		7	0.1748	0.1718	0.1873
		8	0.1258	0.2129	0.2488
		9	0.2417	0.2541	0.4057
		10	0.1753	0.1937	0.2659
		11	0.2076	0.2656	0.3164
		12	0.1462	0.2451	0.1879
		13	0.3407	0.2615	0.3339
		14	0.1881	0.2324	0.2223
		15	0.3080	0.2720	0.3247
		16	0.3465	0.3454	0.3701
KE3	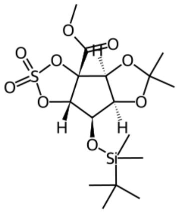	1	0.1498	0.1280	0.2161
		2	0.0778	0.1148	0.0934
		3	0.2081	0.1165	0.2390
		4	0.0680	0.0937	0.1206
		5	0.1165	0.1945	0.1930
		6	0.0971	0.0386	0.1058
		7	0.0693	0.0739	0.0590
		8	0.1283	0.0876	0.1669
		9	0.1260	0.0911	0.1031
		10	0.0710	0.1202	0.1813
		11	0.1107	0.1898	0.1325
		12	**0.2310**	**0.2239**	**0.2471**
		13	0.1095	0.0846	0.1050
		14	0.1440	0.1183	0.1712
		15	0.1032	0.0785	0.1128
		16	0.1264	0.1173	0.1337

continued on next page

continued from previous page

Name	Molecule	Stereoisomer	Single Conformer	Full DFT	ETKDG
NL1B	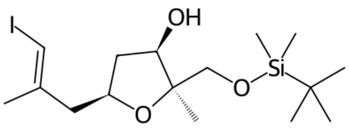	1	**0.3154**	**0.2960**	**0.3648**
		2	0.2963	0.1666	0.2829
		3	0.2406	0.2046	0.2589
		4	0.1293	0.2095	0.2499
NL2A	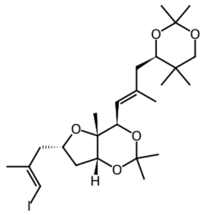	1	0.1225	**0.4289**	**0.4063**
		2	0.1010	0.3676	0.2841
		3	0.1274	0.3156	0.2362
		4	0.1055	0.3161	0.2137
		5	0.0624	0.3338	0.2805
		6	0.0927	0.3419	0.1812
		7	0.0652	0.3211	0.2460
		8	0.1030	0.2980	0.2505
		9	0.1272	0.3362	0.2973
		10	0.1192	0.3664	0.2289
		11	0.1104	0.2846	0.2216
		12	0.2065	0.3992	0.3043
		13	0.1442	0.3844	0.3263
		14	0.1825	0.3366	0.1848
		15	0.1139	0.3647	0.2803
		16	**0.2095**	0.2436	0.2245
NL2B	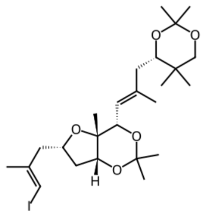	1	0.0675	0.1456	0.1125
		2	0.0630	**0.1556**	0.1371
		3	0.0548	0.0939	0.0771
		4	0.1354	0.1423	0.0930
		5	0.0485	0.1364	0.1575
		6	0.0655	0.1023	0.0641
		7	0.0412	0.1337	0.1651
		8	0.1089	0.1256	0.1118
		9	0.0463	0.1082	0.1191
		10	0.0319	0.1487	0.1132
		11	0.0338	0.0763	0.0700
		12	0.0534	0.1327	0.1323
		13	0.0595	0.1428	0.1088
		14	0.0398	0.1198	0.0705
		15	0.0434	0.1464	**0.1674**
		16	**0.1475**	0.0957	0.0965

continued on next page

continued from previous page

Name	Molecule	Stereoisomer	Single Conformer	Full DFT	ETKDG
		1	0.1624	0.2601	0.3742
		2	0.1664	0.1143	0.1662
		3	0.1476	0.1783	0.2526
		4	0.1612	0.0862	0.1612
		5	**0.3885**	0.5636	0.3392
		6	0.1391	0.1670	0.3367
		7	0.1052	0.4791	0.5046
		8	0.0552	0.1426	0.2131
		9	0.0914	0.2319	0.3308
		10	0.1525	0.1007	0.1525
		11	0.1390	0.2958	0.3535
		12	0.2510	0.2217	0.2510
		13	0.2433	0.5708	0.4711
		14	0.1805	0.1452	0.1351
		15	0.3192	0.4723	0.4402
NP1	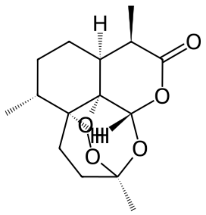	16	0.2398	0.2490	0.3689
		17	0.1166	0.2867	0.4604
		18	0.0509	0.1095	0.1620
		19	0.0746	0.2523	0.2770
		20	0.0874	0.0219	0.0874
		21	0.1999	0.1755	0.2000
		22	0.0716	0.2300	0.2606
		23	0.1457	0.3709	0.4906
		24	0.0949	0.2172	0.3592
		25	0.0491	0.3204	0.3084
		26	0.1203	0.0441	0.1202
		27	0.1065	0.2770	0.3377
		28	0.1229	0.0879	0.1230
		29	0.1397	0.2887	0.3903
		30	0.1158	0.2323	0.2762
		31	0.2989	**0.5958**	**0.5898**
		32	0.3199	0.3235	0.3198

continued on next page

continued from previous page

Name	Molecule	Stereoisomer	Single Conformer	Full DFT	ETKDG
NP2	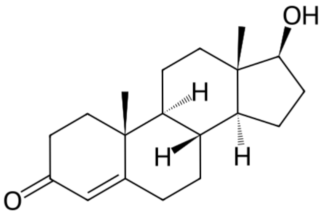	1	0.2356	**0.5114**	**0.4904**
		2	0.0777	0.2565	0.2429
		3	0.2075	0.2991	0.2564
		4	0.0841	0.0797	0.1034
		5	0.1685	0.3102	0.2647
		6	**0.2795**	0.2057	0.2630
		7	0.0852	0.1491	0.1384
		8	0.1267	0.1124	0.1020
		9	0.1282	0.1645	0.1865
		10	0.0460	0.0998	0.0985
		11	0.1838	0.2688	0.3165
		12	0.0401	0.1568	0.1234
		13	0.0604	0.1027	0.1198
		14	0.1354	0.1161	0.1057
		15	0.1176	0.2349	0.2236
		16	0.0644	0.1773	0.1879
		17	0.1114	0.1966	0.2085
		18	0.1430	0.1161	0.1370
		19	0.1051	0.0816	0.0804
		20	0.0500	0.0753	0.0578
		21	0.1590	0.1799	0.1511
		22	0.1828	0.2274	0.2009
		23	0.0820	0.0761	0.0713
		24	0.0667	0.1487	0.0811
		25	0.0550	0.1304	0.1319
		26	0.0706	0.1215	0.1285
		27	0.0953	0.1667	0.1323
		28	0.0689	0.1278	0.0964
		29	0.0737	0.1291	0.1061
		30	0.1219	0.2635	0.2340
		31	0.0646	0.0862	0.1190
		32	0.1754	0.2895	0.2055
NP3A	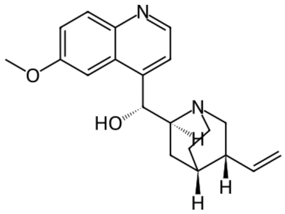	1	**0.3475**	0.3241	**0.3903**
		2	0.1279	0.2320	0.1698
		3	0.0675	**0.3700**	0.3202
		4	0.0523	0.2116	0.2219
		5	0.1248	0.2853	0.3037
		6	0.0778	0.3430	0.2750
		7	0.2726	0.2700	0.2532
		8	0.2329	0.2284	0.2550

continued on next page

continued from previous page

Name	Molecule	Stereoisomer	Single Conformer	Full DFT	ETKDG
NP3B	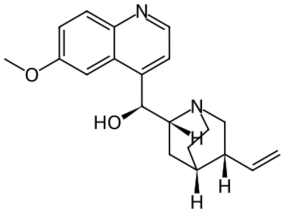	1	0.0828	0.3463	**0.3412**
		2	0.1211	0.3379	0.2632
		3	0.0815	**0.3517**	0.2929
		4	0.1857	0.2375	0.2258
		5	**0.1922**	0.3450	0.3074
		6	0.1797	0.3406	0.3192
		7	0.1203	0.2999	0.2453
		8	0.1480	0.2029	0.2038
NP4	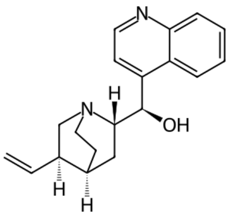	1	**0.2831**	**0.4340**	**0.5545**
		2	0.2621	0.4234	0.4503
		3	0.1626	0.2906	0.2163
		4	0.2318	0.2795	0.3298
		5	0.2468	0.3148	0.3238
		6	0.2269	0.3337	0.4440
		7	0.2301	0.3764	0.2582
		8	0.2066	0.3769	0.5239
NP5	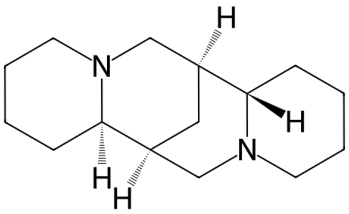	1	0.2624	**0.6013**	0.4663
		2	**0.2715**	0.5171	**0.5619**
OD1	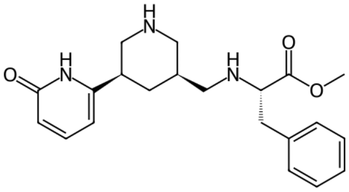	1	0.0816	0.1337	0.0986
		2	0.0586	**0.1705**	**0.1225**
		3	0.0707	0.1351	0.0899
		4	**0.0977**	0.1009	0.0753
TP1	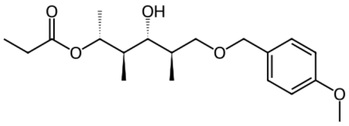	1	0.4391	**0.5651**	**0.4985**
		2	**0.5243**	0.5277	0.4223
		3	0.2850	0.3768	0.3718
		4	0.4684	0.4238	0.3774
		5	0.3858	0.4170	0.3404
		6	0.4043	0.3788	0.3649
		7	0.4036	0.4621	0.4732
		8	0.3045	0.4346	0.3541

continued on next page

continued from previous page

Name	Molecule	Stereoisomer	Single Conformer	Full DFT	ETKDG
TP2	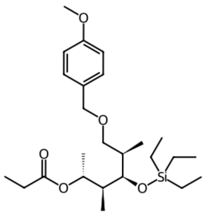	1	0.3275	0.4583	0.4703
		2	0.4382	0.3703	0.3585
		3	0.3446	0.4593	0.3581
		4	0.3941	0.3950	0.4175
		5	0.4209	0.4472	0.3899
		6	0.3168	0.4312	0.4363
		7	**0.4583**	0.4543	**0.4918**
		8	0.2919	**0.5014**	0.4336
TP3	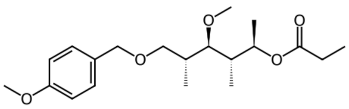	1	0.3817	0.4848	**0.4780**
		2	0.3790	0.3764	0.4064
		3	0.3456	0.4252	0.3394
		4	0.2404	0.3584	0.3786
		5	0.1591	0.3869	0.4342
		6	0.2369	0.4242	0.3924
		7	0.2819	**0.5005**	0.4508
		8	**0.3978**	0.3862	0.4241
TS1	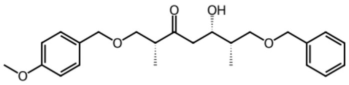	1	**0.3266**	0.2083	0.3963
		2	0.2475	0.2044	0.3462
		3	0.2082	0.2207	0.3711
		4	0.3136	**0.3534**	**0.4041**
TS2	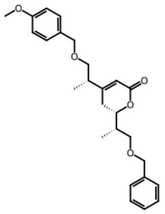	1	0.3797	0.4828	0.4329
		2	0.3808	0.4319	0.4312
		3	0.3531	**0.5123**	**0.4913**
		4	**0.4226**	0.4652	0.4515
TS3B	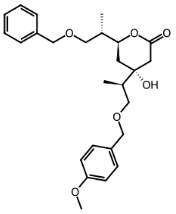	1	0.2342	0.2782	0.2619
		2	**0.3104**	0.2839	0.2699
		3	0.2652	0.3737	0.2788
		4	0.2781	0.2567	0.2940
		5	0.2928	0.2560	0.2559
		6	0.2750	**0.3824**	**0.3262**
		7	0.2920	0.2914	0.2363
		8	0.1270	0.2921	0.2684

D DP5 Analysis of Structural Reassignment Examples

This section contains results of DFT-free DP5 analysis of the 24 structural reassignment examples presented in this paper. For all molecules, conformational analysis was performed using ETKDG algorithm.

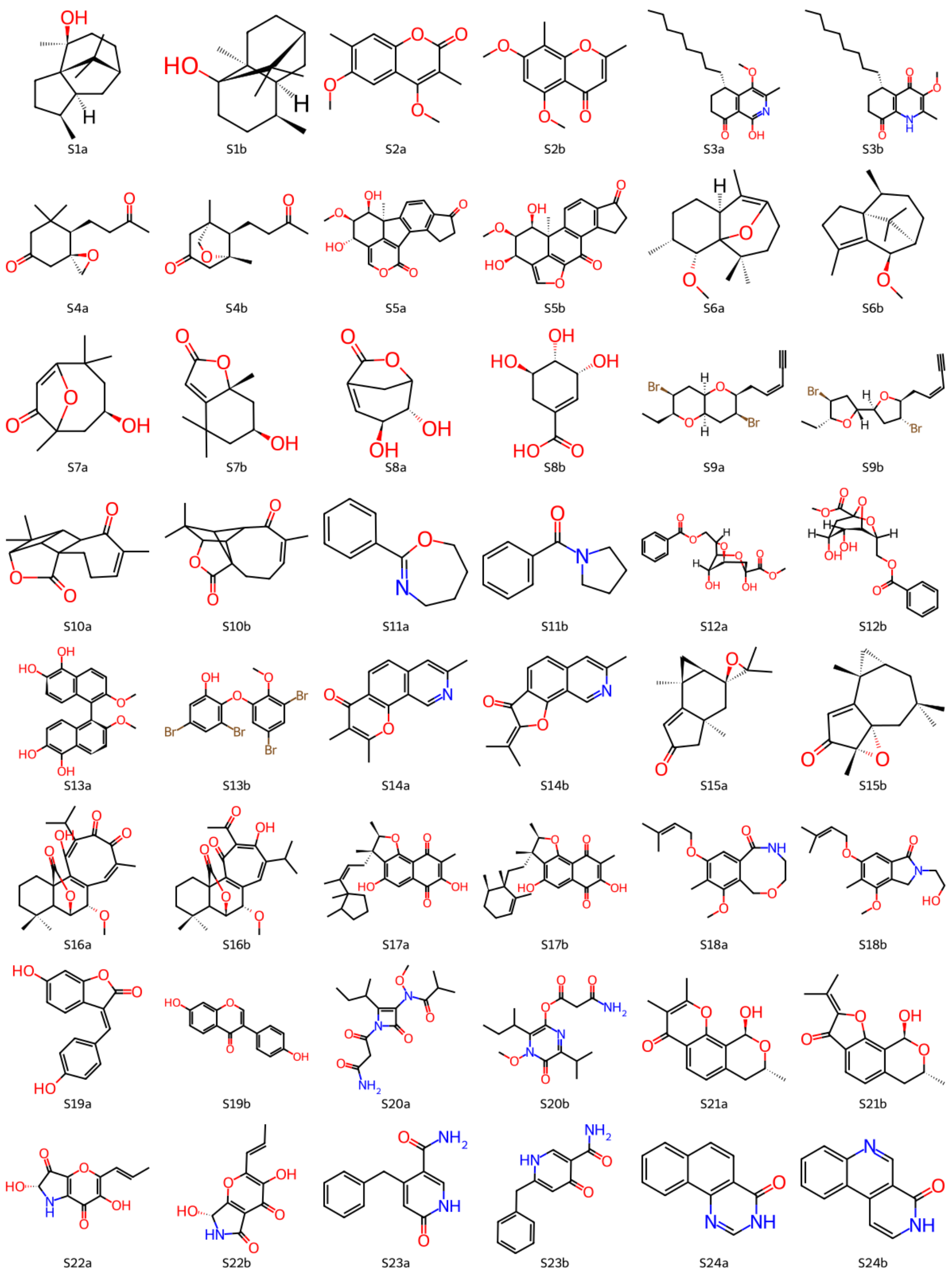

Examples of molecules which had their initial published structures reassigned. Here, letter ‘a’ in the name (e.g., S1**a**) denotes an initial proposal, and letter ‘b’ (e.g., S1**b**) denotes a reassigned structure.

S1

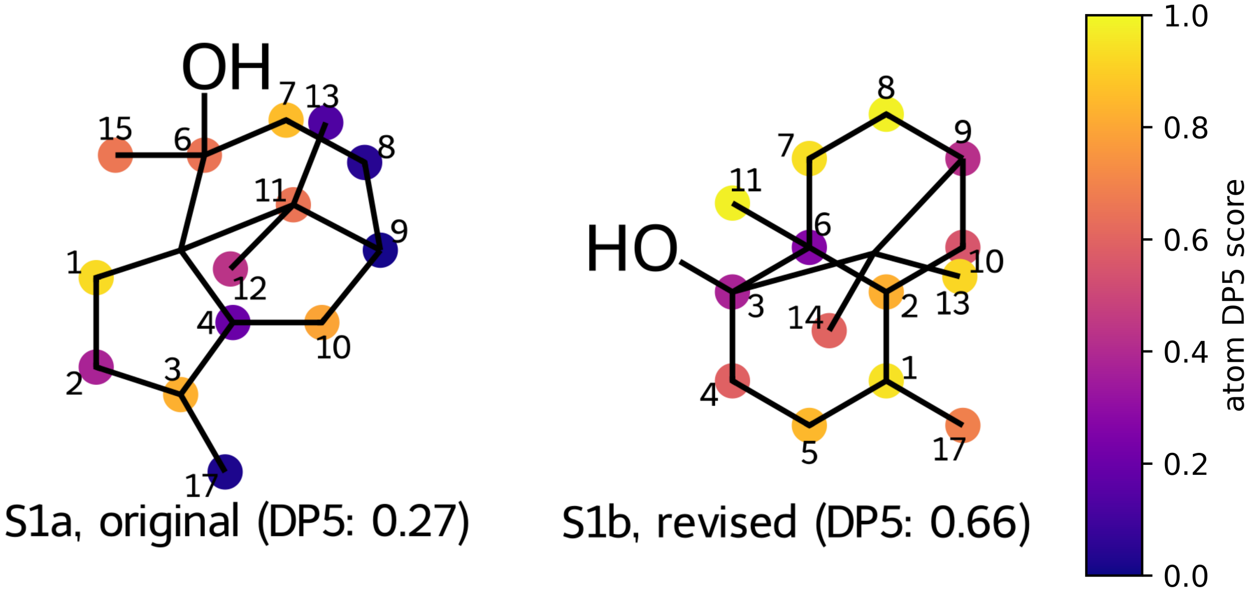

S1a, original proposal

label	calculated	assigned	error	DP5
C1	26.93	26.9	0.03	0.93
C2	31.72	28.1	3.62	0.38
C3	34.22	32.8	1.42	0.83
C4	44.95	40.1	4.85	0.20
C6	76.65	75.2	1.45	0.66
C7	37.86	37.6	0.26	0.86
C8	28.00	24.6	3.40	0.04
C9	47.51	39.1	8.41	0.01
C10	29.46	28.2	1.26	0.80
C11	47.24	43.7	3.54	0.65
C12	23.80	24.4	-0.60	0.43
C13	23.02	20.7	2.32	0.14
C15	28.93	28.6	0.33	0.66
C17	14.07	18.7	-4.63	0.03

S1b, revised proposal

label	calculated	assigned	error	DP5
C1	29.42	28.6	0.82	0.95
C2	42.97	43.7	-0.73	0.82
C3	77.58	75.2	2.38	0.38
C4	37.19	37.6	-0.41	0.59
C5	28.14	28.2	-0.06	0.84
C6	42.96	40.1	2.86	0.27
C7	32.18	32.8	-0.62	0.94
C8	24.17	24.4	-0.23	0.98
C9	41.69	39.1	2.59	0.42
C10	25.54	26.9	-1.36	0.55
C11	19.63	20.7	-1.07	0.98
C13	22.95	24.6	-1.65	0.92
C14	27.88	28.1	-0.22	0.60
C17	18.33	18.7	-0.37	0.69

S2

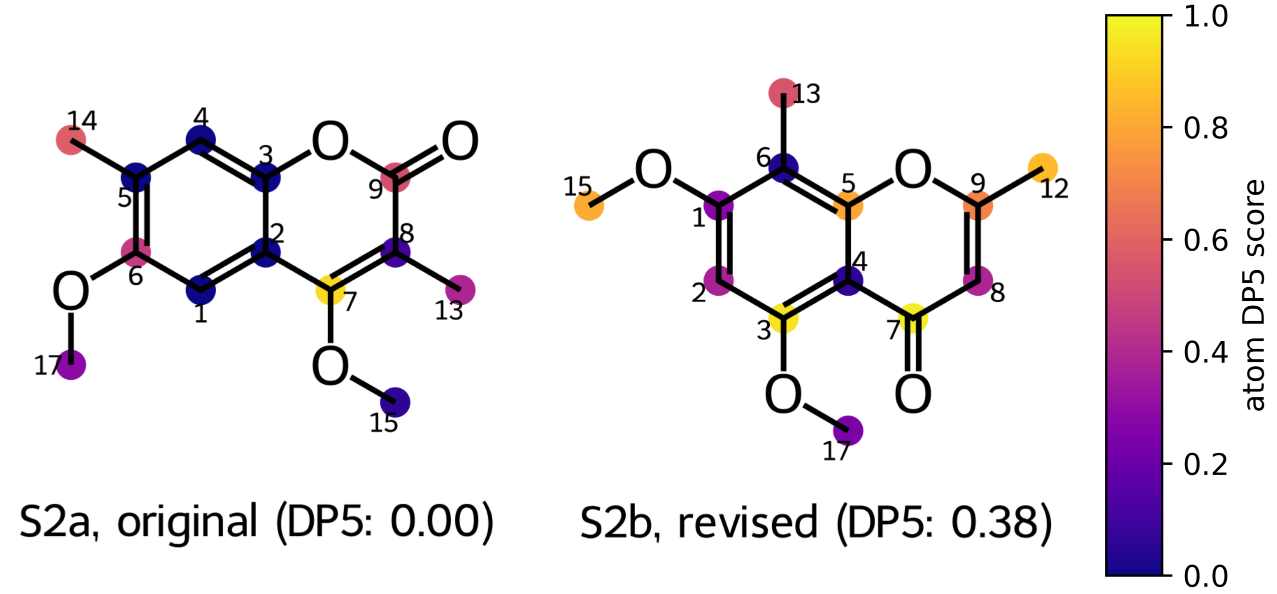

S2a, original proposal

label	calculated	assigned	error	DP5
C1	102.37	91.1	11.27	0.00
C2	113.16	105.3	7.86	0.00
C3	149.31	177.6	-28.29	0.00
C4	120.79	108.0	12.79	0.00
C5	130.63	158.5	-27.87	0.00
C6	153.13	156.5	-3.37	0.46
C7	160.12	160.7	-0.58	0.93
C8	116.57	110.9	5.67	0.10
C9	161.54	162.6	-1.06	0.54
C13	9.46	7.3	2.16	0.39
C14	17.33	19.4	-2.07	0.58
C15	55.34	55.4	-0.06	0.06
C17	52.07	56.0	-3.93	0.29

S2b, revised proposal

label	calculated	assigned	error	DP5
C1	161.36	158.5	2.86	0.28
C2	94.14	91.1	3.04	0.37
C3	161.53	160.7	0.83	0.95
C4	108.25	105.3	2.95	0.06
C5	156.36	156.5	-0.14	0.80
C6	113.56	108.0	5.56	0.04
C7	173.81	177.6	-3.79	0.97
C8	115.73	110.9	4.83	0.38
C9	163.54	162.6	0.94	0.70
C12	19.47	19.4	0.07	0.85
C13	8.59	7.3	1.29	0.55
C15	51.47	56.0	-4.53	0.81
C17	53.75	55.4	-1.65	0.25

S3

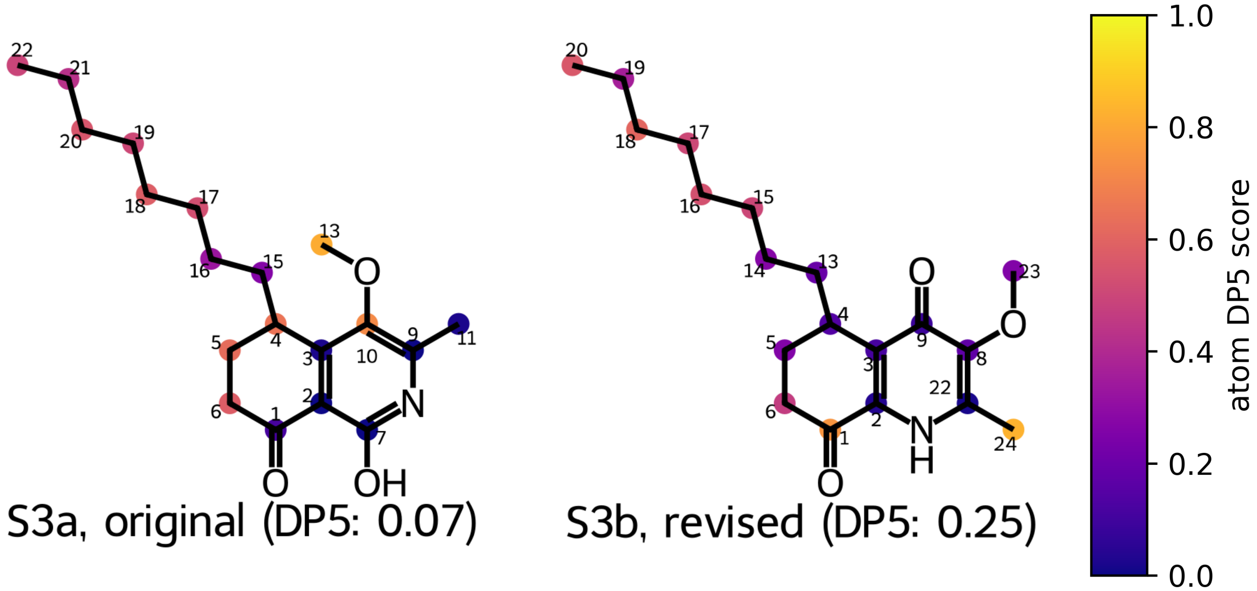

S3a, original proposal

label	calculated	assigned	error	DP5
C1	200.37	194.6	5.77	0.12
C2	109.67	132.2	-22.53	0.00
C3	144.94	139.0	5.94	0.02
C4	32.96	32.2	0.76	0.65
C5	29.59	29.5	0.09	0.63
C6	30.76	30.5	0.26	0.58
C7	162.26	172.8	-10.54	0.00
C9	154.33	138.9	15.43	0.00
C10	148.67	147.5	1.17	0.72
C11	19.33	14.5	4.83	0.02
C13	57.48	59.4	-1.92	0.81
C15	33.82	31.8	2.02	0.27
C16	26.93	24.3	2.63	0.33
C17	30.29	29.6	0.69	0.53
C18	29.38	29.2	0.18	0.58
C19	29.16	28.4	0.76	0.51
C20	31.30	30.3	1.00	0.55
C21	23.43	22.6	0.83	0.43
C22	13.78	14.0	-0.22	0.49

S3b, revised proposal

label	calculated	assigned	error	DP5
C1	190.68	194.6	-3.92	0.75
C2	131.44	138.9	-7.46	0.04
C3	126.55	132.2	-5.65	0.11
C4	33.04	30.5	2.54	0.14
C5	29.08	28.4	0.68	0.26
C6	32.62	32.2	0.42	0.47
C8	144.44	147.5	-3.06	0.17
C9	177.71	172.8	4.91	0.09
C13	34.70	30.3	4.40	0.20
C14	27.22	24.3	2.92	0.27
C15	30.10	29.5	0.60	0.50
C16	29.70	29.6	0.10	0.54
C17	30.24	29.2	1.04	0.50
C18	32.18	31.8	0.38	0.61
C19	24.25	22.6	1.65	0.36
C20	13.72	14.0	-0.28	0.56
C22	130.61	139.0	-8.39	0.02
C23	53.74	59.4	-5.66	0.26
C24	14.85	14.5	0.35	0.83

S4

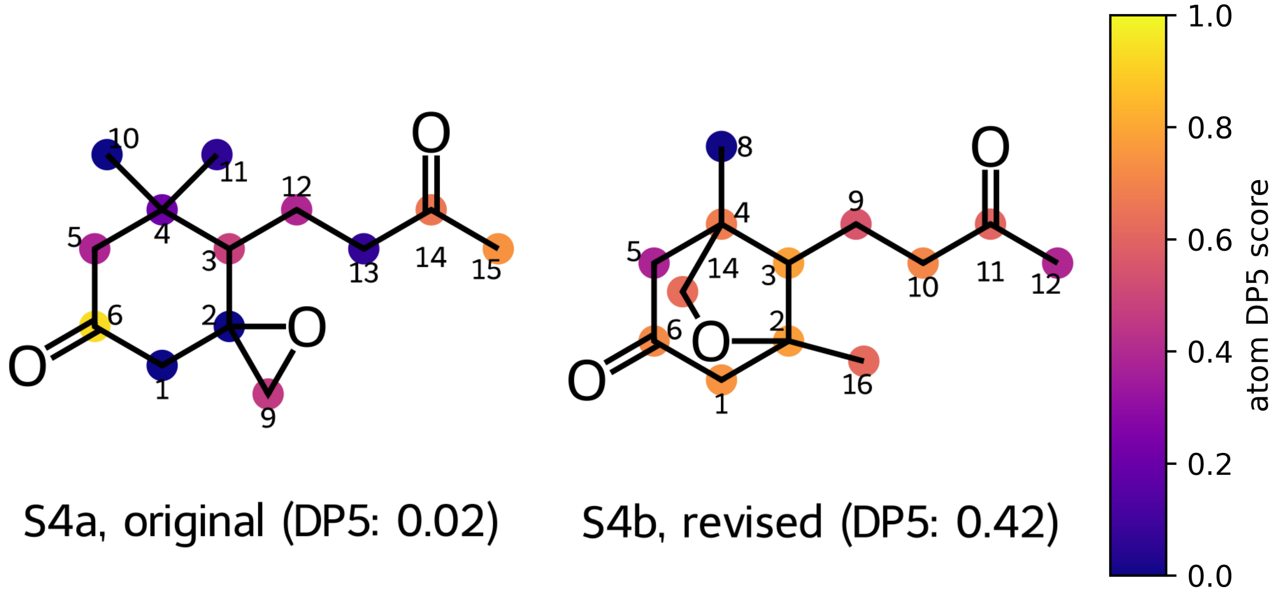

S4a, original proposal				
label	calculated	assigned	error	DP5
C1	43.85	78.3	-34.45	0.00
C2	56.58	83.4	-26.82	0.00
C3	46.80	48.6	-1.80	0.48
C4	40.75	42.6	-1.85	0.21
C5	48.99	49.4	-0.41	0.39
C6	208.65	207.2	1.45	0.94
C9	52.33	53.1	-0.77	0.46
C10	27.93	18.7	9.23	0.00
C11	27.14	24.9	2.24	0.06
C12	23.31	20.8	2.51	0.39
C13	38.46	43.4	-4.94	0.07
C14	210.15	208.7	1.45	0.66
C15	29.61	30.0	-0.39	0.74

S4b, revised proposal				
label	calculated	assigned	error	DP5
C1	46.42	48.6	-2.18	0.75
C2	80.43	83.4	-2.97	0.77
C3	52.37	53.1	-0.73	0.78
C4	45.16	43.4	1.76	0.68
C5	48.84	49.4	-0.56	0.38
C6	208.70	207.2	1.50	0.72
C8	21.87	18.7	3.17	0.00
C9	20.82	20.8	0.02	0.55
C10	42.01	42.6	-0.59	0.71
C11	209.57	208.7	0.87	0.60
C12	29.01	30.0	-0.99	0.39
C14	76.16	78.3	-2.14	0.63
C16	25.25	24.9	0.35	0.62

S5

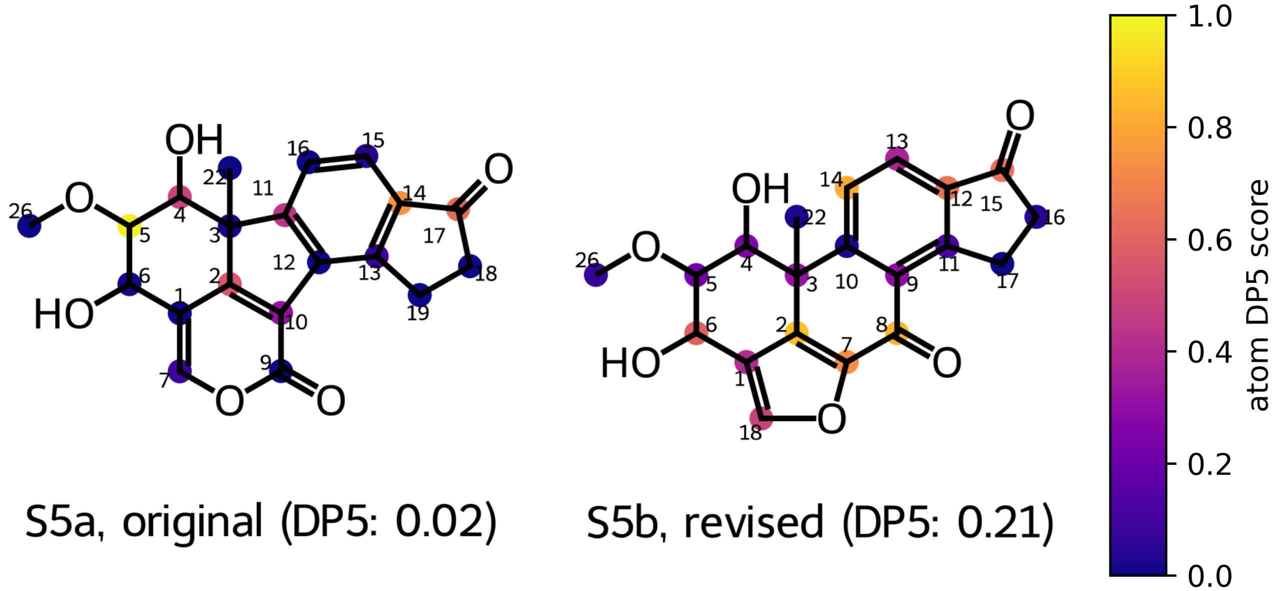

S5a, original proposal

label	calculated	assigned	error	DP5
C1	117.58	129.9	-12.32	0.00
C2	153.78	158.7	-4.92	0.56
C3	52.00	42.4	9.60	0.01
C4	71.23	71.8	-0.57	0.50
C5	81.92	81.7	0.22	0.98
C6	67.47	61.7	5.77	0.01
C7	151.08	145.8	5.28	0.10
C9	161.82	173.5	-11.68	0.00
C10	122.01	122.1	-0.09	0.31
C11	157.82	158.1	-0.28	0.44
C12	132.86	142.4	-9.54	0.00
C13	149.76	145.6	4.16	0.09
C14	136.10	137.0	-0.90	0.76
C15	125.89	127.3	-1.41	0.04
C16	122.18	127.4	-5.22	0.00
C17	201.57	206.7	-5.13	0.63
C18	30.51	36.5	-5.99	0.00
C19	23.95	30.5	-6.55	0.00
C22	21.02	28.5	-7.48	0.00
C26	53.84	60.8	-6.96	0.00

S5b, revised proposal

label	calculated	assigned	error	DP5
C1	125.13	122.1	3.03	0.41
C2	140.77	142.4	-1.63	0.87
C3	44.69	42.4	2.29	0.32
C4	77.90	71.8	6.10	0.26
C5	82.65	81.7	0.95	0.21
C6	62.35	61.7	0.65	0.59
C7	145.91	145.8	0.11	0.74
C8	170.69	173.5	-2.81	0.84
C9	131.42	129.9	1.52	0.30
C10	155.03	158.7	-3.67	0.03
C11	155.49	158.1	-2.61	0.12
C12	135.53	137.0	-1.47	0.65
C13	127.75	127.3	0.45	0.38
C14	127.90	127.4	0.50	0.82
C15	205.03	206.7	-1.67	0.65
C16	32.26	36.5	-4.24	0.05
C17	24.65	30.5	-5.85	0.00
C18	145.58	145.6	-0.02	0.49
C22	23.52	28.5	-4.98	0.02
C26	55.60	60.8	-5.20	0.07

S6

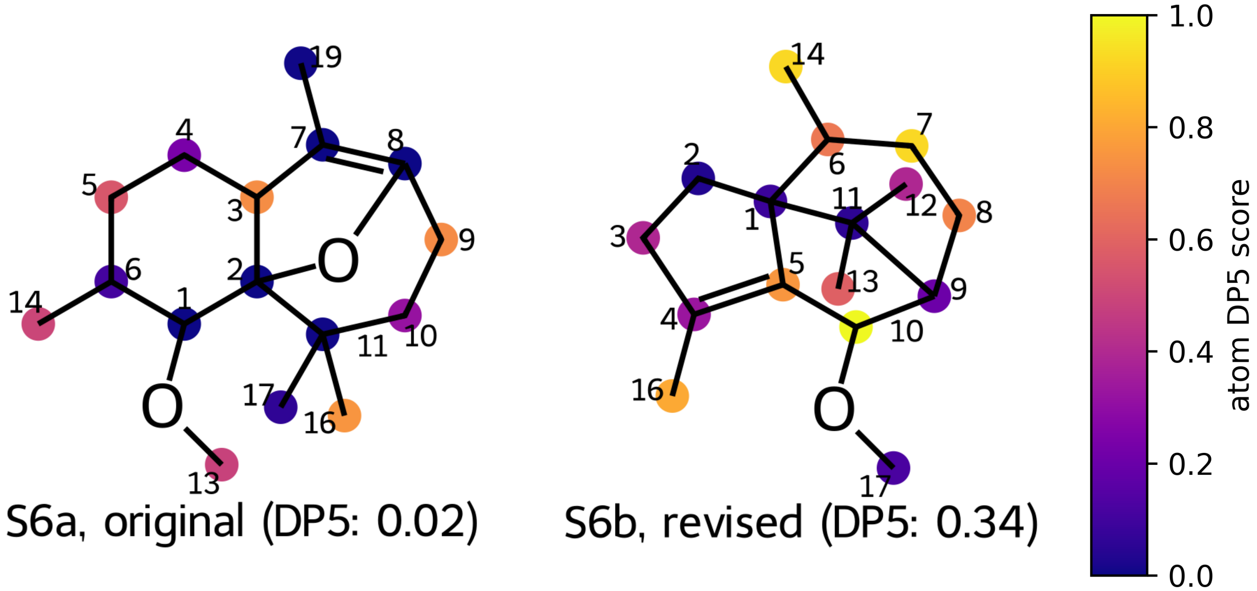

S6a, original proposal				
label	calculated	assigned	error	DP5
C1	78.32	64.9	13.46	0.00
C2	96.36	76.9	19.46	0.00
C3	42.23	42.3	-0.03	0.73
C4	23.46	20.9	2.57	0.24
C5	29.92	28.7	1.18	0.56
C6	32.40	35.5	-3.07	0.11
C7	117.54	136.1	-18.54	0.00
C8	152.41	141.9	10.49	0.00
C9	25.82	27.1	-1.25	0.72
C10	40.17	41.2	-0.99	0.32
C11	43.00	51.0	-8.02	0.01
C13	57.70	57.8	-0.10	0.49
C14	16.66	18.1	-1.42	0.50
C16	24.61	26.2	-1.61	0.75
C17	24.55	22.0	2.50	0.06
C19	7.18	14.5	-7.28	0.00

S6b, revised proposal				
label	calculated	assigned	error	DP5
C1	60.23	64.9	-4.63	0.09
C2	33.99	28.7	5.25	0.03
C3	39.35	41.2	-1.81	0.39
C4	132.42	136.1	-3.66	0.33
C5	142.65	141.9	0.73	0.75
C6	37.12	35.5	1.65	0.67
C7	26.86	27.1	-0.21	0.93
C8	20.45	22.0	-1.60	0.70
C9	49.18	51.0	-1.84	0.20
C10	79.36	76.9	2.46	1.00
C11	47.81	42.3	5.55	0.05
C12	20.07	20.9	-0.82	0.39
C13	24.85	26.2	-1.37	0.59
C14	15.98	18.1	-2.10	0.92
C16	14.55	14.5	0.09	0.81
C17	52.79	57.8	-5.01	0.12

S7

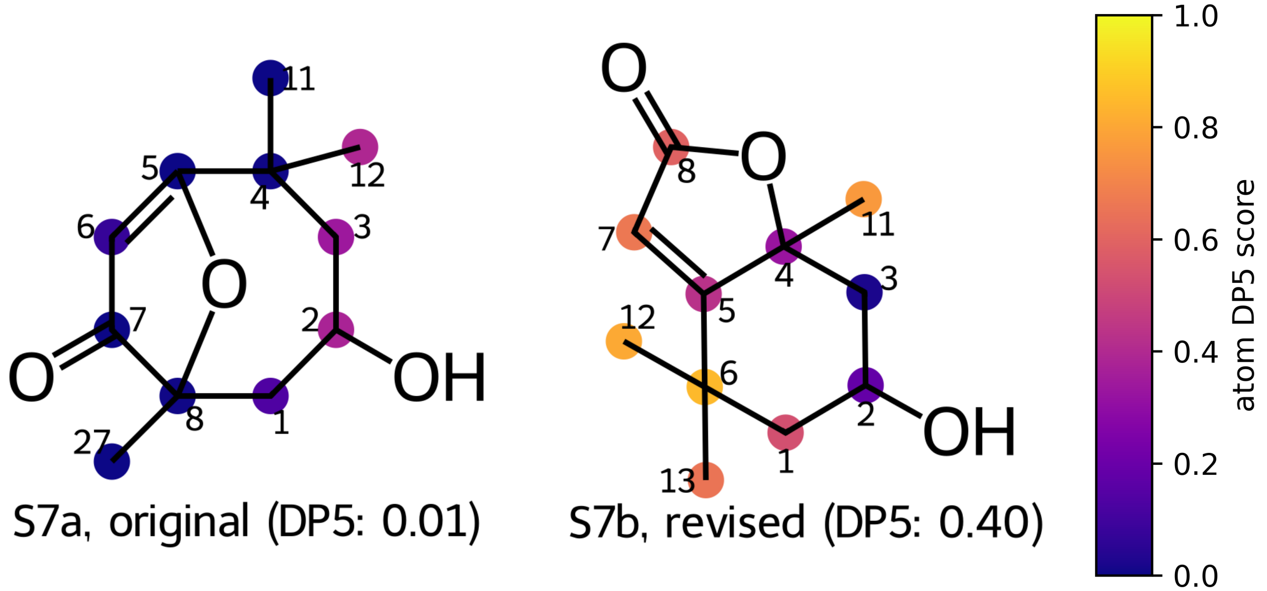

S7a, original proposal

label	calculated	assigned	error	DP5
C1	38.24	36.1	2.14	0.14
C2	66.15	65.3	0.85	0.38
C3	50.36	47.0	3.36	0.34
C4	40.38	45.7	-5.32	0.01
C5	188.95	171.5	17.45	0.00
C6	110.87	112.5	-1.63	0.08
C7	206.03	183.5	22.53	0.00
C8	78.49	86.9	-8.41	0.00
C11	23.10	30.9	-7.80	0.00
C12	22.43	26.6	-4.17	0.39
C27	17.34	27.3	-9.96	0.00

S7b, revised proposal

label	calculated	assigned	error	DP5
C1	44.69	47.0	-2.31	0.54
C2	67.92	65.3	2.62	0.19
C3	38.40	45.7	-7.30	0.02
C4	79.82	86.9	-7.08	0.33
C5	180.04	183.5	-3.46	0.43
C6	37.09	36.1	0.99	0.85
C7	117.19	112.5	4.69	0.67
C8	169.32	171.5	-2.18	0.60
C11	25.38	26.6	-1.22	0.76
C12	25.21	27.3	-2.09	0.65
C13	27.94	30.9	-2.96	0.81

S8

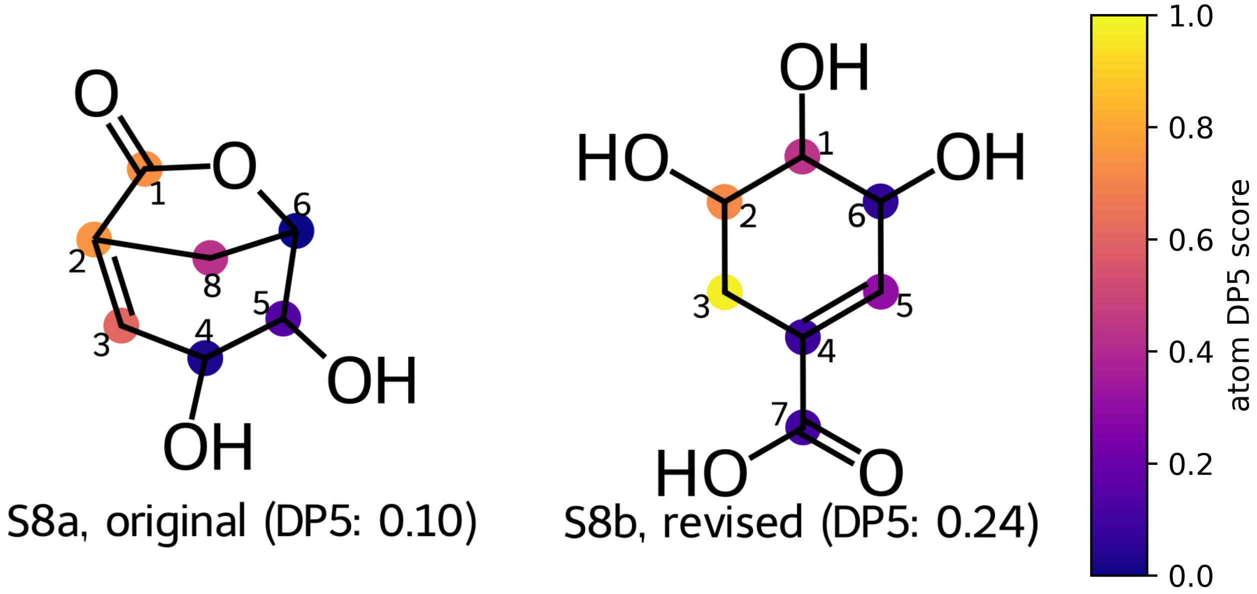

S8a, original proposal				
label	calculated	assigned	error	DP5
C1	174.58	174.9	-0.32	0.73
C2	131.96	130.9	1.06	0.75
C3	138.64	135.5	3.14	0.60
C4	76.20	66.7	9.50	0.02
C5	77.15	71.9	5.25	0.14
C6	73.96	66.2	7.76	0.00
C8	29.23	32.4	-3.17	0.43

S8b, revised proposal				
label	calculated	assigned	error	DP5
C1	70.97	71.9	-0.93	0.43
C2	66.61	66.7	-0.09	0.72
C3	32.75	32.4	0.35	0.97
C4	129.14	130.9	-1.76	0.08
C5	137.31	135.5	1.81	0.30
C6	63.71	66.2	-2.49	0.06
C7	167.13	174.9	-7.77	0.11

S9

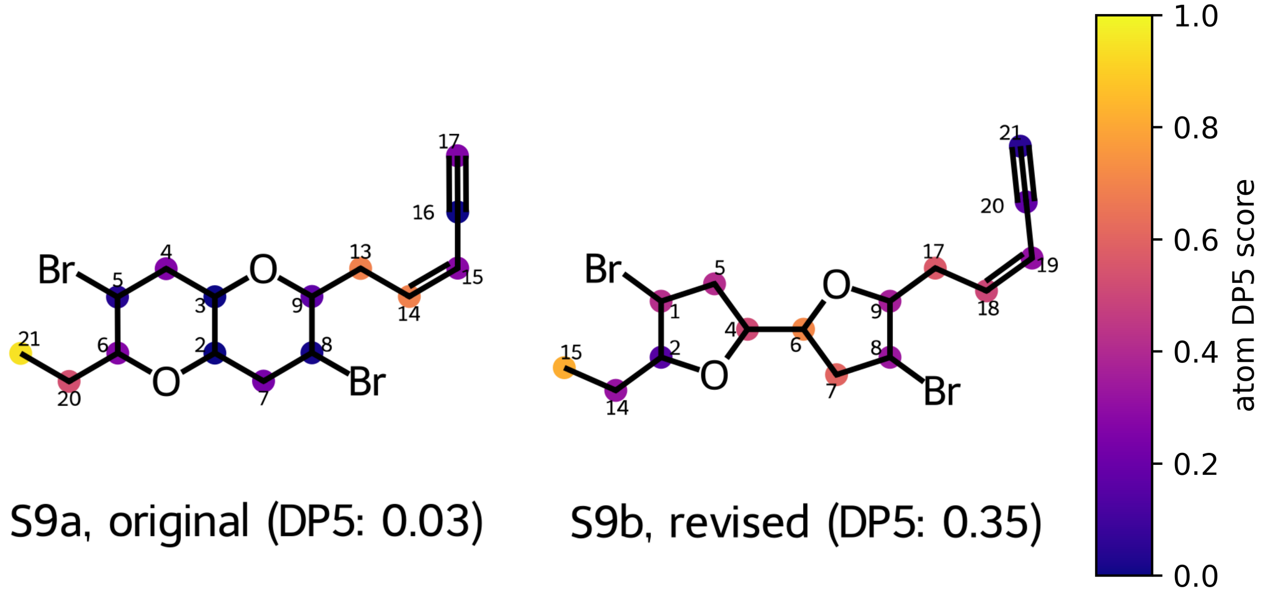

S9a, original proposal				
label	calculated	assigned	error	DP5
C2	67.03	89.0	-21.97	0.00
C3	67.13	79.5	-12.37	0.00
C4	36.75	39.2	-2.45	0.27
C5	57.28	49.2	8.08	0.04
C6	78.06	80.1	-2.04	0.27
C7	36.36	39.0	-2.64	0.24
C8	59.58	48.8	10.78	0.01
C9	77.77	80.2	-2.43	0.17
C13	35.41	34.8	0.61	0.69
C14	144.66	140.1	4.56	0.69
C15	107.69	111.4	-3.71	0.28
C16	76.39	86.7	-10.31	0.00
C17	82.16	82.7	-0.54	0.26
C20	28.86	27.0	1.86	0.53
C21	10.25	10.3	-0.05	0.95

S9b, revised proposal				
label	calculated	assigned	error	DP5
C1	54.98	48.8	6.18	0.42
C2	85.55	89.0	-3.45	0.15
C4	77.22	80.2	-2.98	0.51
C5	38.38	39.0	-0.62	0.41
C6	77.87	80.1	-2.23	0.71
C7	39.68	39.2	0.48	0.60
C8	54.88	49.2	5.68	0.33
C9	84.64	86.7	-2.06	0.36
C14	29.83	27.0	2.83	0.32
C15	9.77	10.3	-0.53	0.82
C17	36.59	34.8	1.79	0.57
C18	142.01	140.1	1.91	0.50
C19	107.95	111.4	-3.45	0.31
C20	77.27	79.5	-2.23	0.18
C21	81.03	82.7	-1.67	0.05

S10

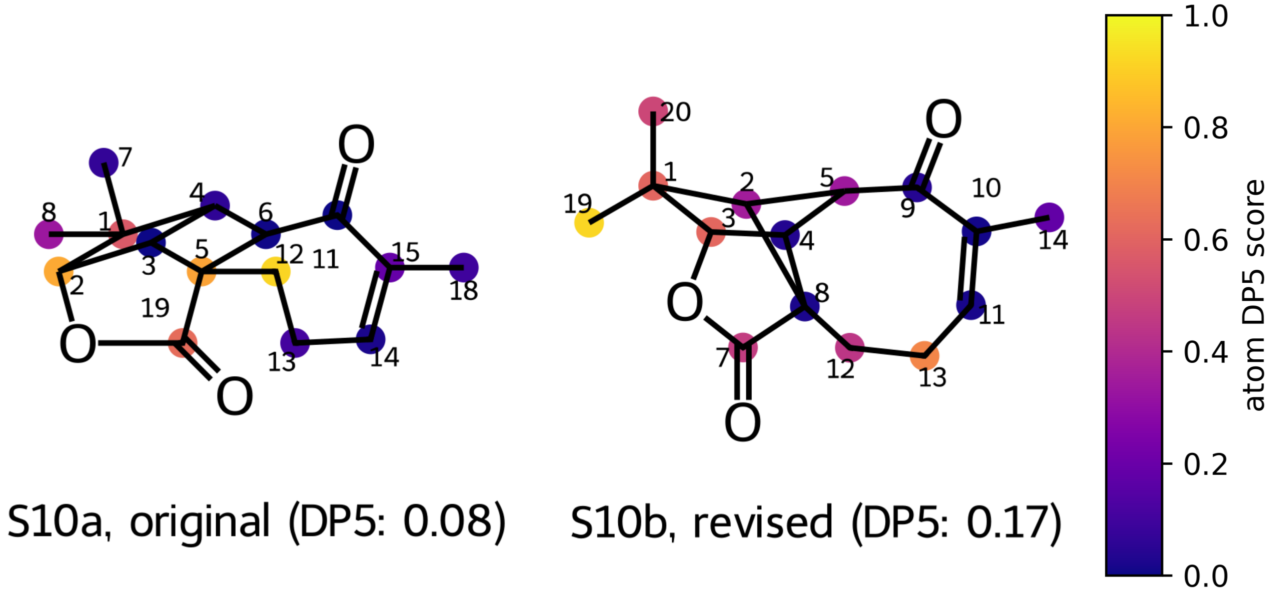

S10a, original proposal

label	calculated	assigned	error	DP5
C1	39.43	41.7	-2.30	0.57
C2	82.65	84.1	-1.47	0.81
C3	45.63	54.5	-8.85	0.00
C4	47.98	54.4	-6.39	0.06
C5	59.77	62.6	-2.80	0.79
C6	56.87	62.8	-5.90	0.01
C7	24.58	22.5	2.13	0.07
C8	18.72	22.1	-3.35	0.33
C11	197.45	211.6	-14.13	0.00
C12	29.31	28.5	0.78	0.92
C13	25.76	22.7	3.03	0.11
C14	136.50	130.9	5.56	0.02
C15	139.84	135.1	4.77	0.19
C18	18.75	22.0	-3.25	0.10
C19	176.97	177.3	-0.30	0.63

S10b, revised proposal

label	calculated	assigned	error	DP5
C1	41.47	41.7	-0.26	0.61
C2	57.63	54.5	3.15	0.35
C3	82.12	84.1	-2.00	0.61
C4	47.36	54.4	-7.01	0.04
C5	60.38	62.6	-2.19	0.34
C7	176.75	177.3	-0.52	0.47
C8	70.25	62.8	7.48	0.02
C9	206.66	211.6	-4.92	0.04
C10	141.71	130.9	10.77	0.01
C11	140.35	135.1	5.28	0.02
C12	21.90	22.0	-0.10	0.44
C13	27.85	28.5	-0.68	0.71
C14	18.83	22.1	-3.24	0.18
C19	22.89	22.7	0.16	0.50
C20	21.34	22.5	-1.11	0.92

S11

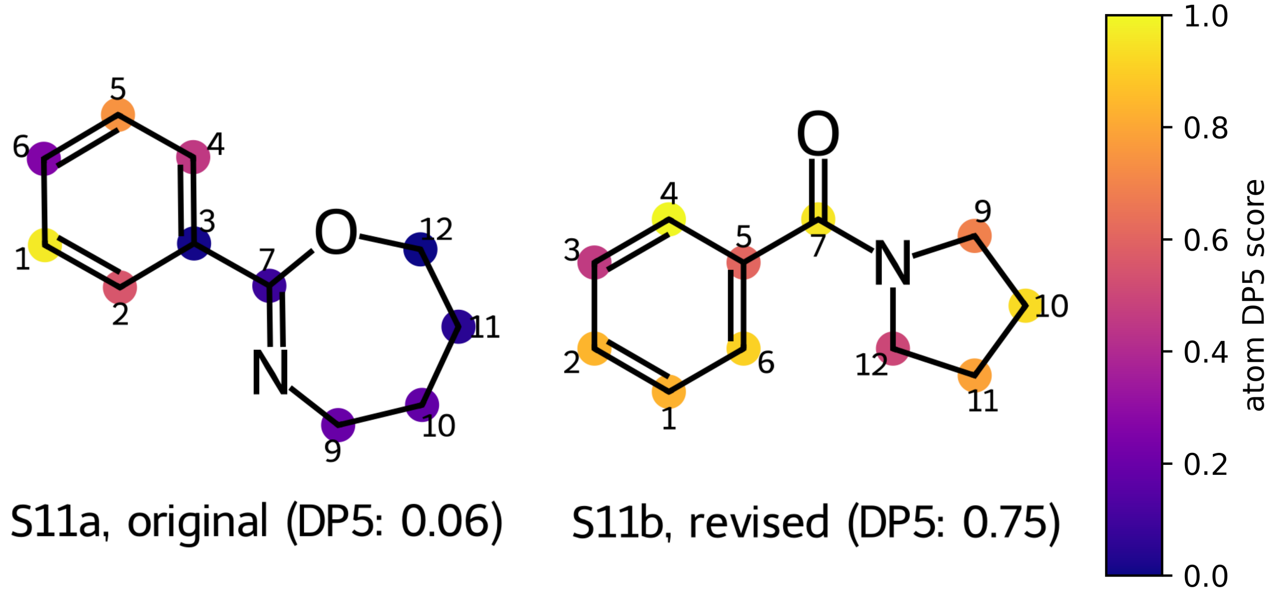

S11a, original proposal				
label	calculated	assigned	error	DP5
C1	127.49	128.3	-0.81	0.97
C2	130.81	129.8	1.01	0.55
C3	132.26	137.3	-5.04	0.01
C4	127.05	128.3	-1.25	0.45
C5	126.40	127.1	-0.70	0.75
C6	130.49	129.8	0.69	0.26
C7	165.10	169.8	-4.70	0.09
C9	50.13	46.2	3.93	0.20
C10	27.46	24.5	2.96	0.18
C11	30.98	26.4	4.58	0.05
C12	67.52	49.6	17.92	0.00

S11b, revised proposal				
label	calculated	assigned	error	DP5
C1	127.26	128.3	-1.04	0.83
C2	129.19	129.8	-0.61	0.84
C3	128.96	129.8	-0.84	0.46
C4	128.55	128.3	0.25	1.00
C5	135.78	137.3	-1.52	0.61
C6	123.52	127.1	-3.58	0.91
C7	169.04	169.8	-0.76	0.95
C9	45.99	46.2	-0.21	0.50
C10	25.85	24.5	1.35	0.79
C11	27.04	26.4	0.64	0.93
C12	46.98	49.6	-2.62	0.69

S12

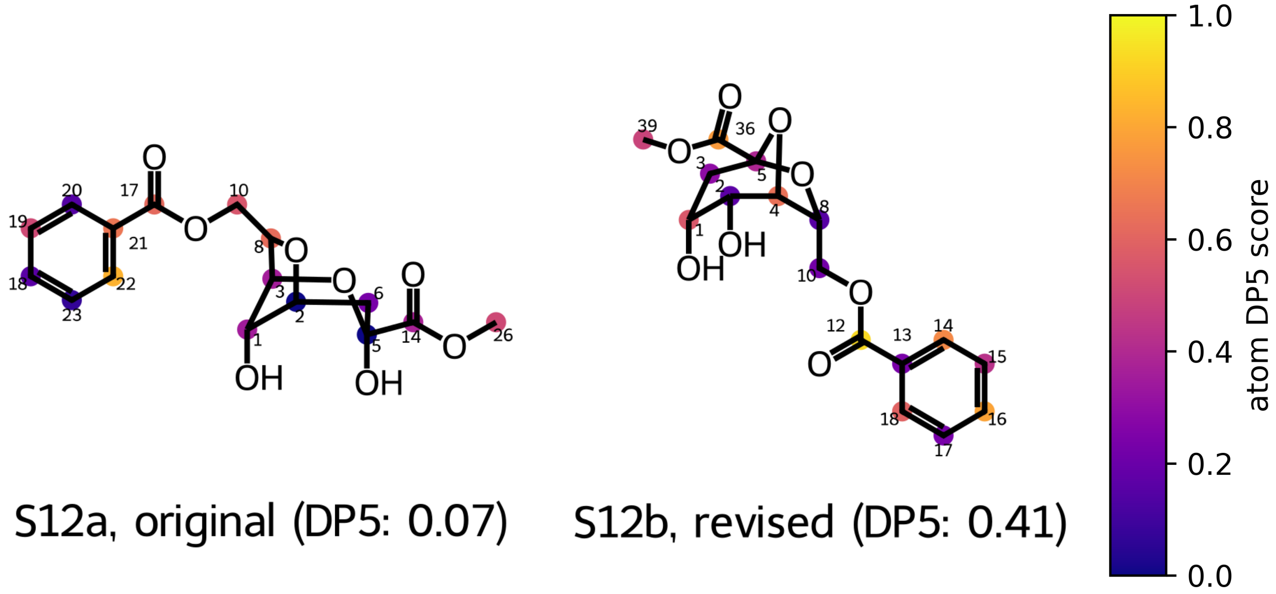

S12a, original proposal				
label	calculated	assigned	error	DP5
C1	71.45	69.5	1.95	0.35
C2	77.76	65.8	11.96	0.00
C3	79.71	78.4	1.31	0.36
C5	92.82	104.9	-12.08	0.00
C6	38.71	40.4	-1.69	0.24
C8	80.96	81.0	-0.04	0.63
C10	64.95	65.1	-0.15	0.55
C14	173.79	169.6	4.19	0.37
C17	166.55	167.9	-1.35	0.62
C18	132.81	131.4	1.41	0.16
C19	127.24	130.6	-3.36	0.07
C20	130.55	130.6	-0.05	0.83
C21	129.07	129.5	-0.43	0.66
C22	132.95	134.2	-1.25	0.15
C23	127.66	129.5	-1.84	0.51
C26	50.66	53.3	-2.64	0.51

S12b, revised proposal				
label	calculated	assigned	error	DP5
C1	65.86	65.8	0.06	0.56
C2	67.27	69.5	-2.23	0.17
C3	39.28	40.4	-1.12	0.29
C4	77.46	78.4	-0.94	0.64
C5	105.25	104.9	0.35	0.39
C8	79.14	81.0	-1.86	0.15
C10	63.06	65.1	-2.04	0.22
C12	167.99	167.9	0.09	0.93
C13	127.44	130.6	-3.16	0.24
C14	132.42	131.4	1.02	0.72
C15	127.48	129.5	-2.02	0.43
C16	132.95	134.2	-1.25	0.79
C17	126.80	129.5	-2.70	0.24
C18	130.06	130.6	-0.54	0.59
C36	169.06	169.6	-0.54	0.77
C39	50.87	53.3	-2.43	0.49

S13

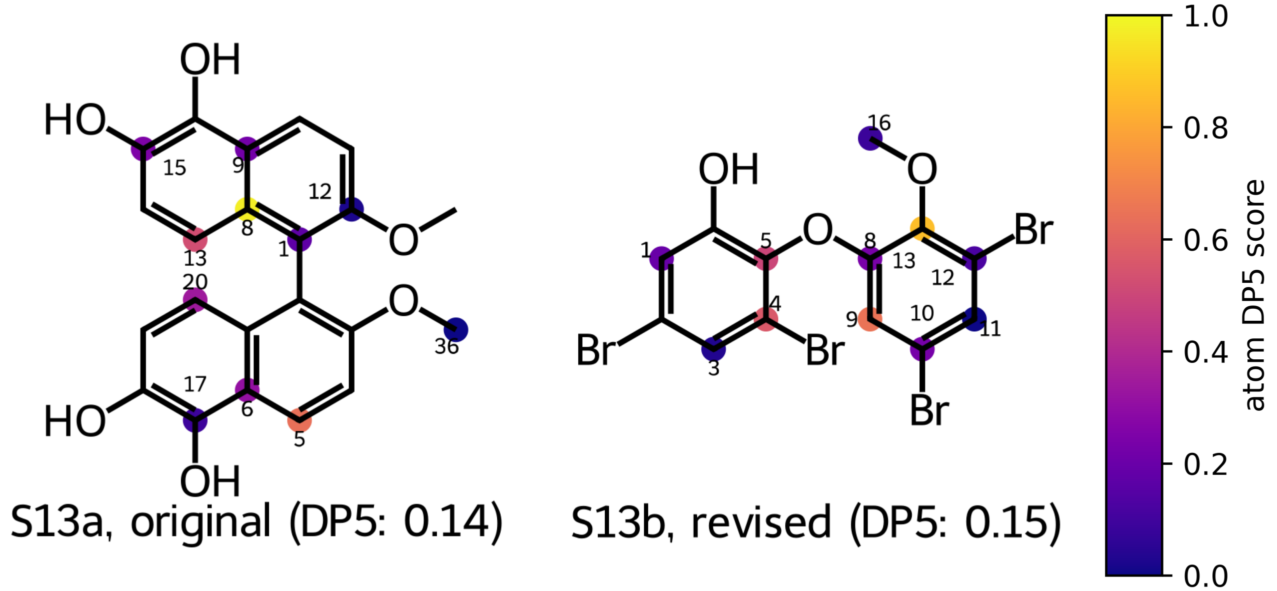

S13a, original proposal				
label	calculated	assigned	error	DP5
C1	125.21	120.2	5.01	0.17
C5	125.60	127.4	-1.80	0.64
C6	116.40	118.2	-1.80	0.31
C8	130.32	130.2	0.12	0.98
C9	116.30	117.4	-1.10	0.23
C12	159.71	150.5	9.21	0.02
C13	118.09	118.5	-0.41	0.53
C15	138.36	138.5	-0.14	0.25
C17	140.78	145.0	-4.22	0.09
C20	118.53	119.9	-1.37	0.34
C36	53.26	61.5	-8.24	0.00

S13b, revised proposal				
label	calculated	assigned	error	DP5
C1	113.14	117.4	-4.26	0.20
C3	123.87	118.5	5.37	0.03
C4	128.32	127.4	0.92	0.56
C5	139.72	138.5	1.22	0.50
C8	147.26	150.5	-3.24	0.24
C9	117.24	118.2	-0.96	0.65
C10	129.29	130.2	-0.91	0.23
C11	128.43	119.9	8.53	0.00
C12	127.16	120.2	6.96	0.12
C13	145.11	145.0	0.11	0.86
C16	50.73	61.5	-10.77	0.09

S14

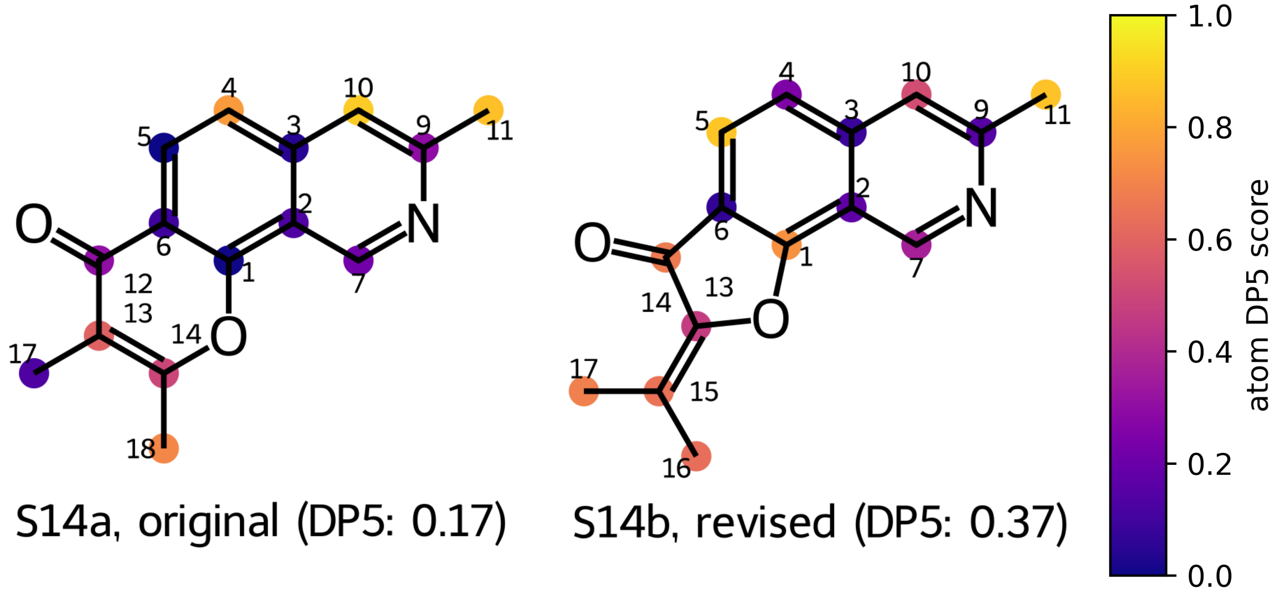

S14a, original proposal

label	calculated	assigned	error	DP5
C1	152.08	145.6	6.48	0.01
C2	113.79	114.6	-0.81	0.14
C3	134.79	141.3	-6.51	0.04
C4	123.83	124.1	-0.27	0.77
C5	130.09	133.7	-3.61	0.00
C6	118.67	119.3	-0.63	0.09
C7	152.41	146.2	6.21	0.22
C9	160.35	156.7	3.65	0.29
C10	117.33	119.5	-2.17	0.89
C11	24.23	24.7	-0.47	0.87
C12	175.12	182.1	-6.98	0.30
C13	120.17	120.5	-0.33	0.60
C14	161.63	164.0	-2.37	0.50
C17	12.67	17.5	-4.83	0.13
C18	19.21	20.4	-1.19	0.71

S14b, revised proposal

label	calculated	assigned	error	DP5
C1	162.33	164.0	-1.67	0.74
C2	107.99	114.6	-6.61	0.17
C3	136.51	141.3	-4.79	0.09
C4	122.96	120.5	2.46	0.25
C5	127.28	124.1	3.18	0.88
C6	120.92	119.5	1.42	0.07
C7	150.88	146.2	4.68	0.36
C9	161.54	156.7	4.84	0.15
C10	118.92	119.3	-0.38	0.53
C11	24.30	24.7	-0.40	0.87
C13	146.71	145.6	1.11	0.47
C14	177.67	182.1	-4.43	0.67
C15	134.79	133.7	1.09	0.65
C16	15.82	17.5	-1.68	0.63
C17	19.49	20.4	-0.91	0.69

S15

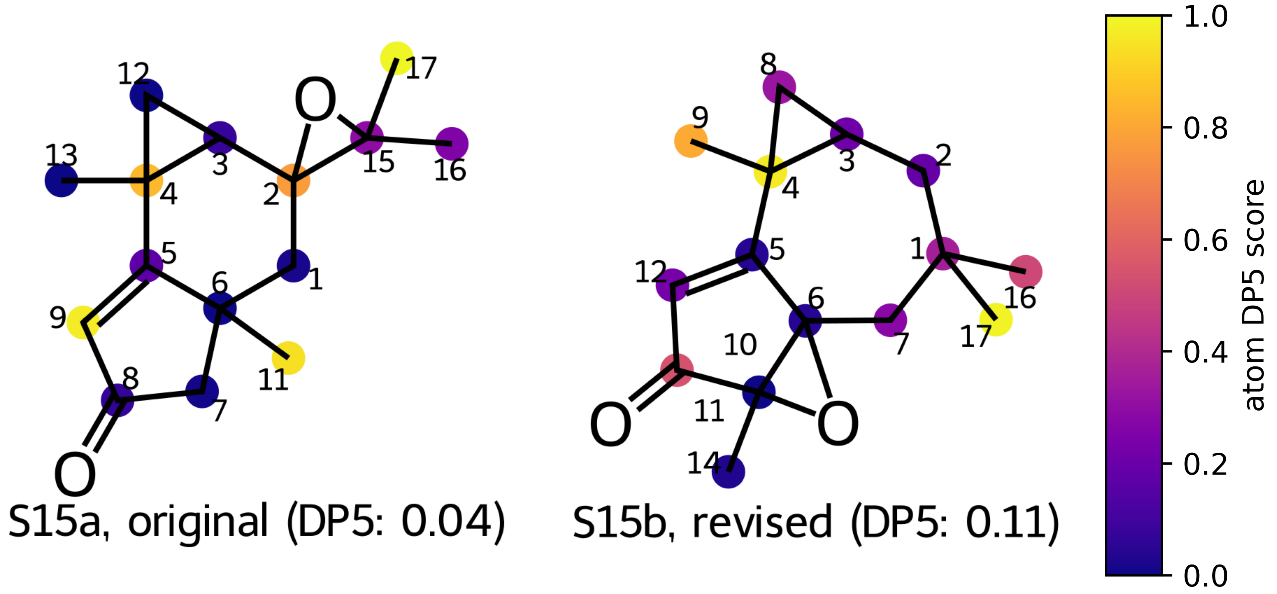

S15a, original proposal

label	calculated	assigned	error	DP5
C1	38.38	34.2	4.18	0.02
C2	61.17	66.5	-5.33	0.77
C3	20.87	21.3	-0.43	0.08
C4	26.23	28.9	-2.67	0.85
C5	187.96	180.0	7.96	0.17
C6	43.54	34.5	9.04	0.00
C7	48.06	42.7	5.36	0.01
C8	203.51	200.1	3.41	0.07
C9	131.87	128.5	3.37	0.97
C11	26.02	26.5	-0.48	0.94
C12	19.28	8.4	10.88	0.00
C13	22.84	30.8	-7.96	0.00
C15	58.83	60.8	-1.97	0.30
C16	19.75	19.5	0.25	0.26
C17	21.40	23.3	-1.90	0.99

S15b, revised proposal

label	calculated	assigned	error	DP5
C1	38.54	34.5	4.04	0.36
C2	38.89	42.7	-3.81	0.19
C3	18.99	19.5	-0.51	0.21
C4	22.20	26.5	-4.30	0.97
C5	172.53	180.0	-7.47	0.04
C6	66.72	66.5	0.22	0.04
C7	36.13	34.2	1.93	0.28
C8	20.61	21.3	-0.69	0.33
C9	23.25	23.3	-0.05	0.81
C10	62.78	60.8	1.98	0.00
C11	194.86	200.1	-5.24	0.54
C12	132.69	128.5	4.19	0.23
C14	10.45	8.4	2.05	0.03
C16	27.56	30.8	-3.24	0.51
C17	27.97	28.9	-0.93	0.99

S16

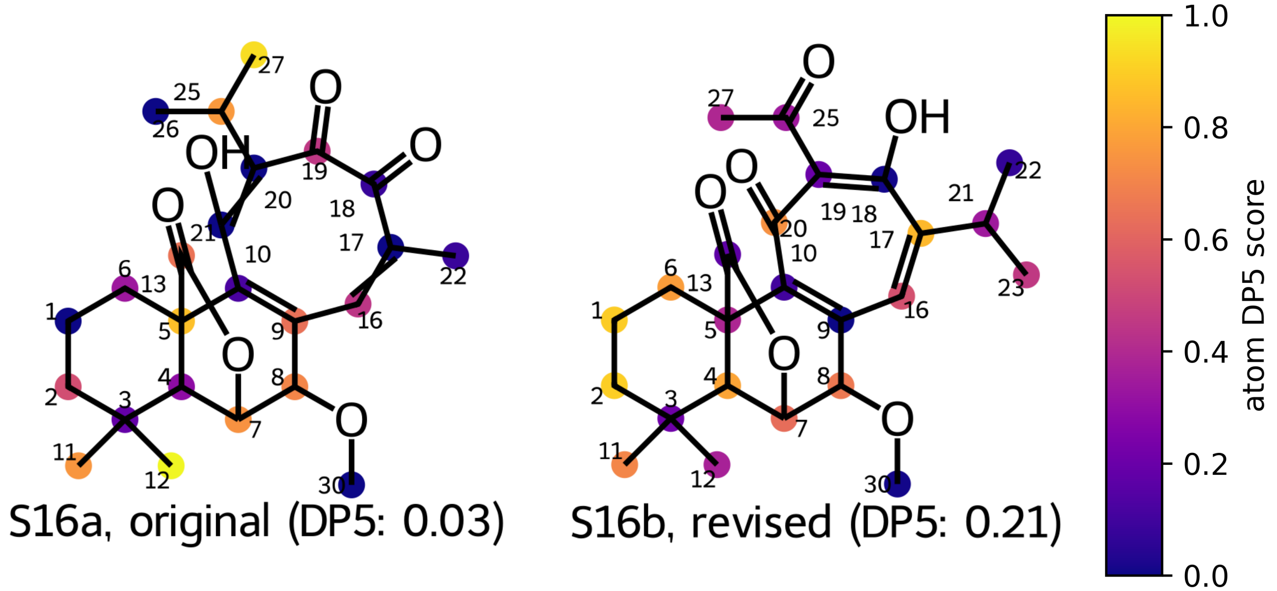

S16a, original proposal

label	calculated	assigned	error	DP5
C1	18.63	28.1	-9.47	0.00
C2	37.02	38.1	-1.08	0.52
C3	34.82	31.3	3.52	0.19
C4	50.01	45.8	4.21	0.30
C5	51.35	50.2	1.15	0.87
C6	23.44	26.4	-2.96	0.34
C7	75.60	73.0	2.60	0.75
C8	84.01	79.5	4.51	0.71
C9	130.79	132.0	-1.21	0.64
C10	121.65	119.8	1.85	0.12
C11	19.94	22.3	-2.36	0.76
C12	28.99	30.2	-1.21	1.00
C13	182.45	175.7	6.75	0.65
C16	132.53	132.1	0.43	0.44
C17	137.44	146.6	-9.16	0.00
C18	198.99	203.1	-4.11	0.08
C19	195.28	189.1	6.18	0.45
C20	132.78	149.1	-16.32	0.00
C21	153.80	175.4	-21.60	0.00
C22	20.01	21.9	-1.89	0.08
C25	30.48	31.3	-0.82	0.77
C26	16.35	22.8	-6.45	0.00
C27	18.05	18.6	-0.55	0.93
C30	51.40	59.2	-7.80	0.00

S16b, revised proposal

label	calculated	assigned	error	DP5
C1	21.28	22.3	-1.02	0.90
C2	38.68	38.1	0.58	0.90
C3	34.77	31.3	3.47	0.21
C4	49.32	50.2	-0.88	0.80
C5	48.33	45.8	2.53	0.39
C6	25.84	26.4	-0.56	0.78
C7	75.68	73.0	2.68	0.63
C8	80.05	79.5	0.55	0.67
C9	135.10	149.1	-14.00	0.00
C10	143.17	146.6	-3.43	0.10
C11	31.47	31.3	0.17	0.71
C12	21.38	21.9	-0.52	0.37
C13	183.09	175.7	7.39	0.17
C16	127.95	132.0	-4.05	0.54
C17	133.87	132.1	1.77	0.85
C18	161.66	175.4	-13.74	0.01
C19	114.35	119.8	-5.45	0.20
C20	189.12	189.1	0.02	0.74
C21	33.58	30.2	3.38	0.34
C22	17.65	22.8	-5.15	0.07
C23	18.18	18.6	-0.42	0.46
C25	197.59	203.1	-5.51	0.34
C27	24.19	28.1	-3.91	0.39
C30	53.53	59.2	-5.67	0.01

S17

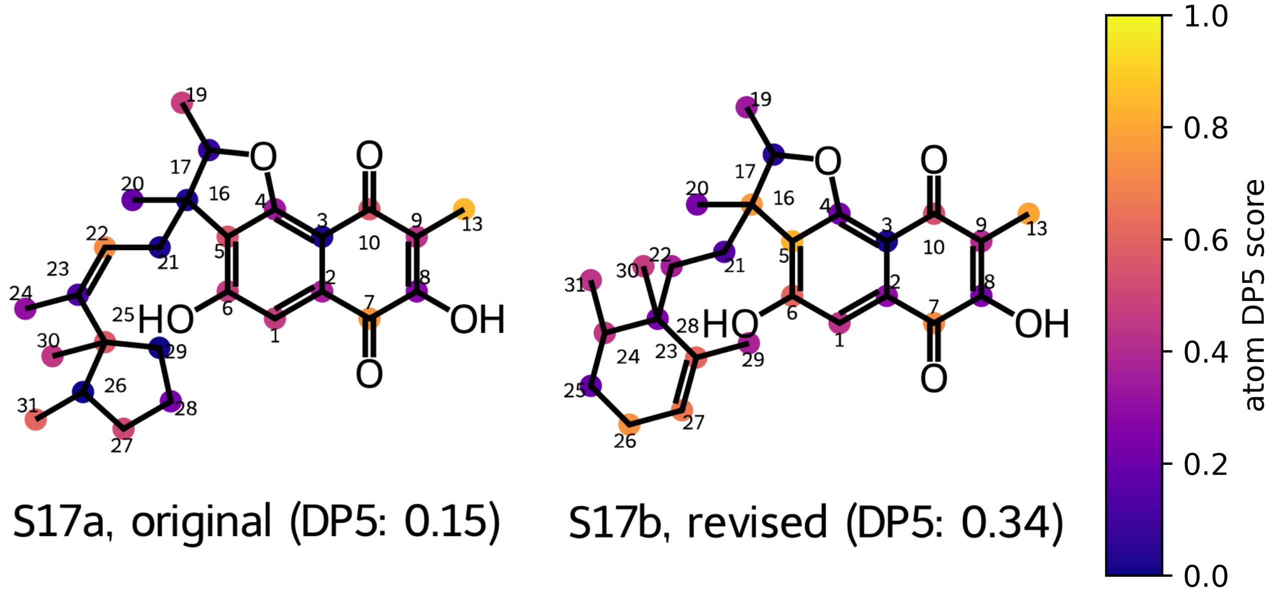

S17a, original proposal

label	calculated	assigned	error	DP5
C1	107.35	107.9	-0.55	0.46
C2	131.12	131.6	-0.48	0.36
C3	111.81	107.9	3.91	0.03
C4	163.89	159.9	3.99	0.35
C5	124.86	123.9	0.96	0.55
C6	155.01	157.7	-2.69	0.47
C7	179.76	180.4	-0.64	0.75
C8	154.46	153.6	0.86	0.28
C9	123.18	120.3	2.88	0.44
C10	182.51	182.9	-0.39	0.56
C13	7.66	8.6	-0.94	0.85
C16	46.51	39.7	6.81	0.04
C17	88.10	86.3	1.80	0.07
C19	15.24	15.1	0.14	0.48
C20	20.86	18.8	2.06	0.19
C21	39.00	32.8	6.20	0.02
C22	128.65	127.3	1.35	0.72
C23	146.59	138.9	7.69	0.12
C24	21.75	19.7	2.05	0.31
C25	48.79	46.0	2.79	0.56
C26	41.36	26.6	14.76	0.00
C27	32.62	31.0	1.62	0.51
C28	22.85	25.1	-2.25	0.23
C29	38.76	30.5	8.26	0.00
C30	21.29	20.9	0.39	0.45
C31	15.34	15.7	-0.36	0.61

S17b, revised proposal

label	calculated	assigned	error	DP5
C1	106.81	107.9	-1.09	0.43
C2	131.38	131.6	-0.22	0.30
C3	112.12	107.9	4.22	0.02
C4	164.77	159.9	4.87	0.23
C5	125.78	127.3	-1.52	0.84
C6	155.68	157.7	-2.02	0.61
C7	179.83	180.4	-0.57	0.73
C8	154.67	153.6	1.07	0.25
C9	123.34	120.3	3.04	0.40
C10	182.60	182.9	-0.30	0.54
C13	7.87	8.6	-0.73	0.79
C16	45.24	46.0	-0.76	0.76
C17	88.89	86.3	2.59	0.05
C19	14.12	15.1	-0.98	0.35
C20	21.94	20.9	1.04	0.23
C21	35.72	31.0	4.72	0.14
C22	32.26	30.5	1.76	0.36
C23	43.32	39.7	3.62	0.24
C24	34.93	32.8	2.13	0.45
C25	28.93	26.6	2.33	0.19
C26	25.95	25.1	0.85	0.74
C27	124.52	123.9	0.62	0.67
C28	141.45	138.9	2.55	0.61
C29	19.17	18.8	0.37	0.37
C30	19.75	19.7	0.05	0.47
C31	16.53	15.7	0.83	0.44

S18

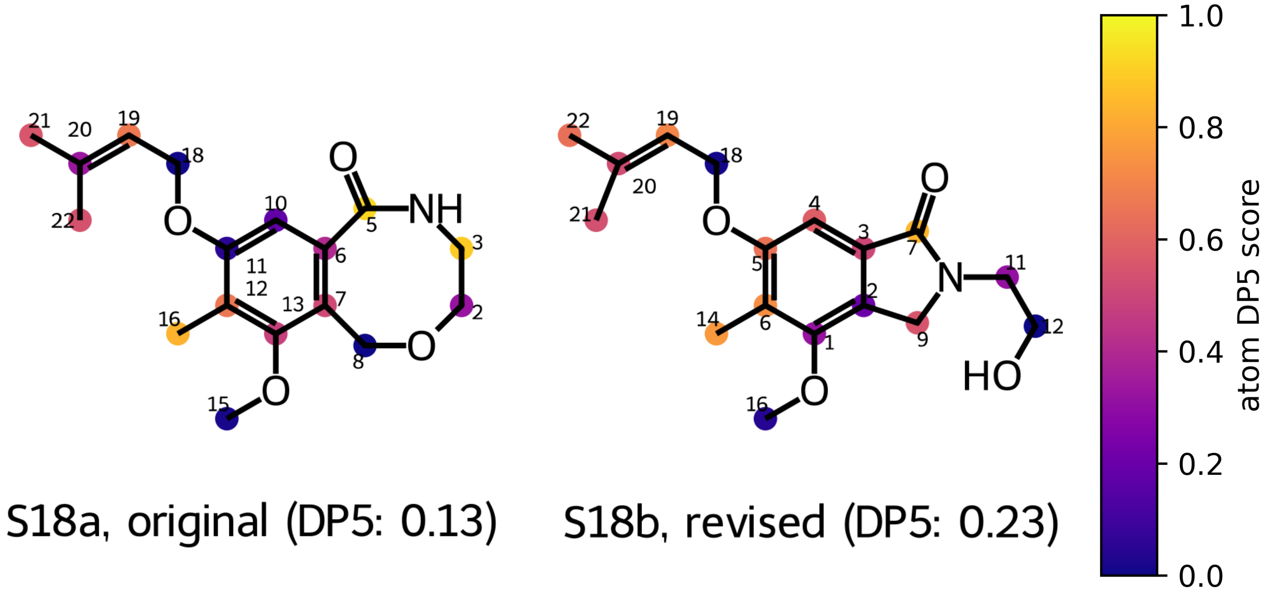

S18a, original (DP5: 0.13)   S18b, revised (DP5: 0.23)

S18a, original proposal

label	calculated	assigned	error	DP5
C2	71.32	68.7	2.62	0.32
C3	45.02	45.1	-0.08	0.89
C5	166.48	167.5	-1.02	0.92
C6	134.45	132.2	2.25	0.40
C7	127.77	125.1	2.67	0.51
C8	63.35	49.2	14.15	0.00
C10	105.68	99.7	5.98	0.18
C11	156.52	152.4	4.12	0.05
C12	124.86	121.8	3.06	0.66
C13	160.77	158.7	2.07	0.48
C15	57.86	56.4	1.46	0.02
C16	10.52	10.2	0.32	0.83
C18	63.78	59.7	4.08	0.01
C19	122.22	120.5	1.72	0.67
C20	135.87	138.0	-2.13	0.33
C21	22.49	26.0	-3.51	0.55
C22	19.14	18.5	0.64	0.54

S18b, revised proposal

label	calculated	assigned	error	DP5
C1	156.02	152.4	3.62	0.32
C2	124.10	120.5	3.60	0.20
C3	131.43	132.2	-0.77	0.50
C4	99.24	99.7	-0.46	0.58
C5	157.90	158.7	-0.80	0.65
C6	125.44	125.1	0.34	0.74
C7	166.98	167.5	-0.52	0.84
C9	47.51	49.2	-1.69	0.55
C11	47.03	45.1	1.93	0.31
C12	63.28	68.7	-5.42	0.00
C14	10.89	10.2	0.69	0.76
C16	57.34	56.4	0.94	0.04
C18	62.95	59.7	3.25	0.01
C19	123.48	121.8	1.68	0.71
C20	137.21	138.0	-0.79	0.52
C21	19.16	18.5	0.66	0.53
C22	23.03	26.0	-2.97	0.64

S19

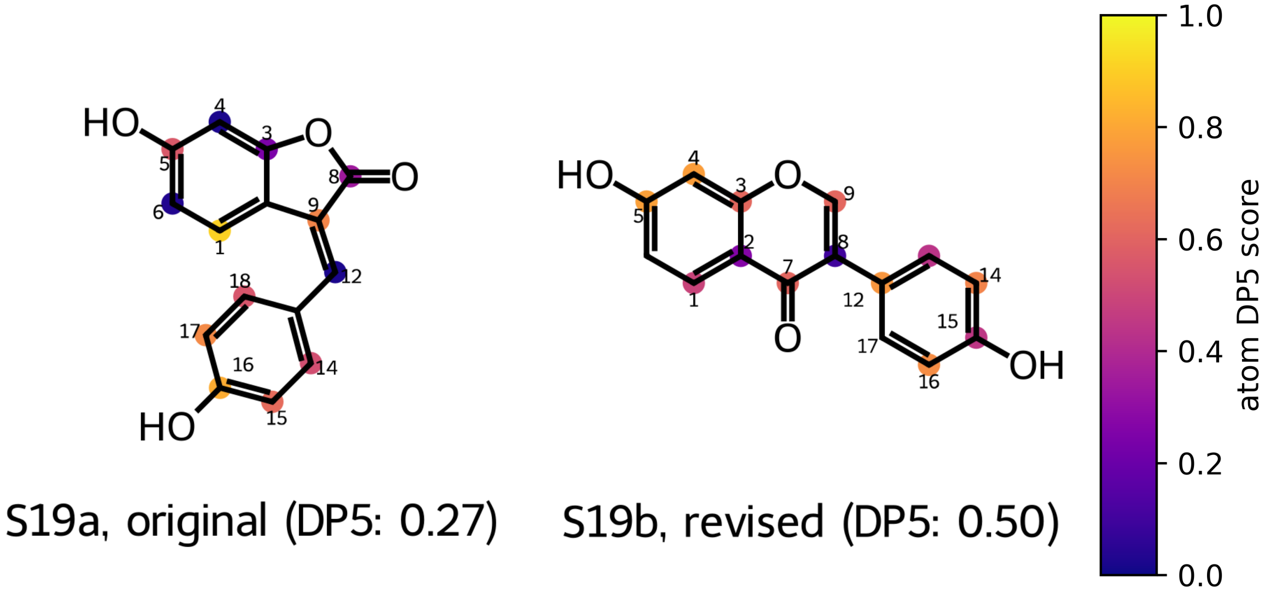

S19a, original proposal

label	calculated	assigned	error	DP5
C1	123.94	122.6	1.34	0.91
C3	157.73	162.8	-5.07	0.24
C4	96.01	102.2	-6.19	0.02
C5	156.69	157.2	-0.51	0.57
C6	111.30	116.6	-5.30	0.03
C8	170.81	174.8	-3.99	0.34
C9	126.09	123.6	2.49	0.71
C12	141.89	152.9	-11.01	0.02
C14	129.44	130.1	-0.66	0.55
C15	115.10	115.3	-0.20	0.72
C16	154.99	157.6	-2.61	0.81
C17	112.75	115.0	-2.25	0.62
C18	128.49	127.3	1.19	0.53

S19b, revised proposal

label	calculated	assigned	error	DP5
C1	131.08	127.3	3.78	0.49
C2	117.62	116.6	1.02	0.27
C3	158.66	157.6	1.06	0.62
C4	101.59	102.2	-0.61	0.77
C5	161.39	162.8	-1.41	0.78
C7	174.03	174.8	-0.77	0.61
C8	126.64	122.6	4.04	0.10
C9	153.82	152.9	0.92	0.61
C12	124.08	123.6	0.48	0.75
C14	113.42	115.3	-1.88	0.73
C15	156.85	157.2	-0.35	0.45
C16	113.42	115.0	-1.58	0.70
C17	132.98	130.1	2.88	0.44

S20

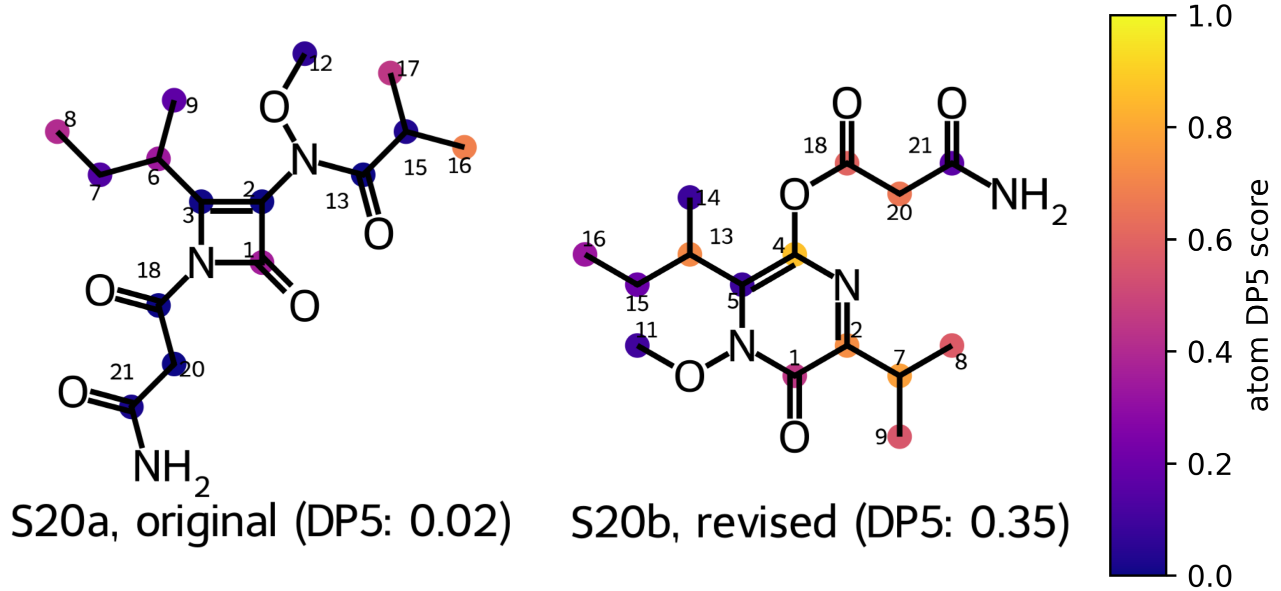

S20a, original proposal

label	calculated	assigned	error	DP5
C1	168.86	168.0	0.86	0.36
C2	114.61	134.9	-20.29	0.00
C3	150.88	135.0	15.88	0.00
C6	40.11	41.1	-0.99	0.34
C7	25.97	27.5	-1.53	0.14
C8	10.62	12.6	-1.98	0.40
C9	18.07	19.9	-1.83	0.17
C12	55.64	64.1	-8.46	0.06
C13	177.41	166.0	11.41	0.00
C15	36.39	30.7	5.69	0.03
C16	17.86	18.2	-0.34	0.69
C17	18.60	19.9	-1.30	0.45
C18	164.25	152.0	12.25	0.00
C20	39.31	33.7	5.61	0.00
C21	165.77	161.5	4.27	0.02

S20b, revised proposal

label	calculated	assigned	error	DP5
C1	148.27	152.0	-3.73	0.45
C2	167.13	166.0	1.13	0.73
C4	138.43	135.0	3.43	0.87
C5	126.98	134.9	-7.92	0.09
C7	32.01	30.7	1.31	0.78
C8	18.17	18.2	-0.03	0.57
C9	18.38	19.9	-1.52	0.56
C11	56.95	64.1	-7.15	0.10
C13	36.07	33.7	2.37	0.72
C14	16.49	19.9	-3.41	0.09
C15	26.56	27.5	-0.94	0.20
C16	11.57	12.6	-1.03	0.33
C18	167.27	168.0	-0.73	0.60
C20	37.22	41.1	-3.88	0.66
C21	161.68	161.5	0.18	0.16

S21

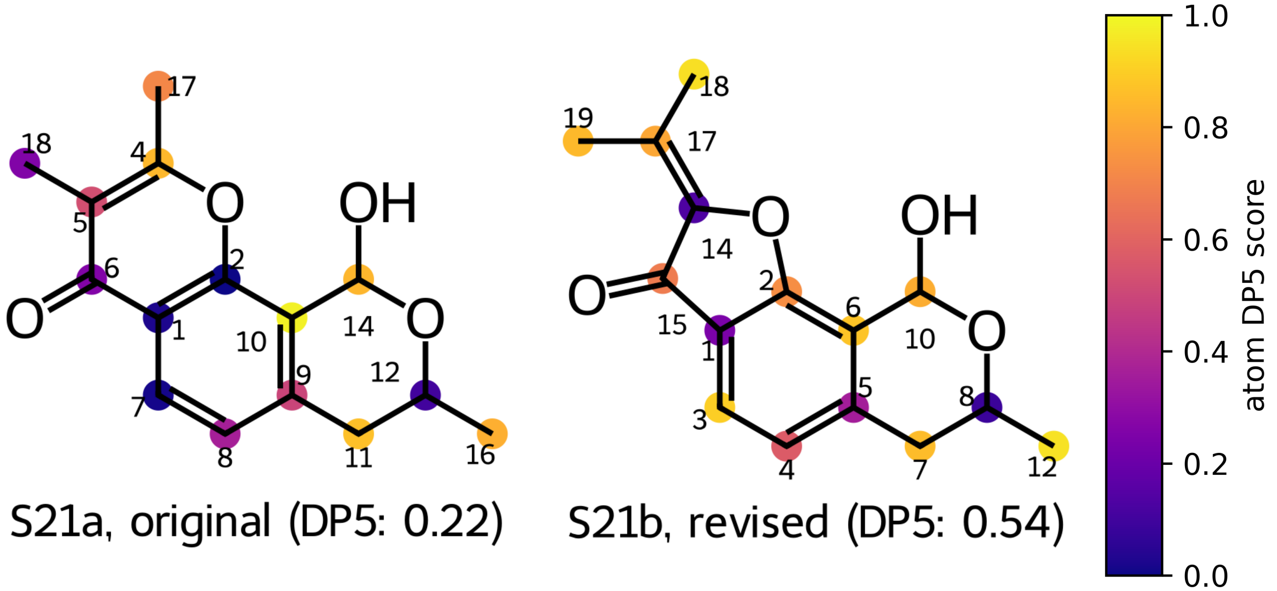

S21a, original proposal

label	calculated	assigned	error	DP5
C1	120.22	119.1	1.12	0.02
C2	153.35	144.5	8.85	0.00
C4	161.34	162.5	-1.16	0.85
C5	120.98	122.5	-1.52	0.53
C6	175.04	182.5	-7.46	0.26
C7	128.44	131.1	-2.66	0.01
C8	126.54	122.7	3.84	0.37
C9	145.62	142.0	3.62	0.49
C10	121.30	122.0	-0.70	0.98
C11	37.27	36.5	0.77	0.86
C12	62.94	62.2	0.74	0.10
C14	90.33	87.5	2.83	0.84
C16	19.99	20.1	-0.11	0.82
C17	18.74	21.0	-2.26	0.71
C18	12.65	17.0	-4.35	0.26

S21b, revised proposal

label	calculated	assigned	error	DP5
C1	121.93	122.0	-0.07	0.24
C2	162.23	162.5	-0.27	0.73
C3	125.58	122.7	2.88	0.90
C4	124.53	122.5	2.03	0.57
C5	145.52	142.0	3.52	0.36
C6	117.75	119.1	-1.35	0.88
C7	37.43	36.5	0.93	0.86
C8	62.86	62.2	0.66	0.09
C10	90.99	87.5	3.49	0.82
C12	20.98	21.0	-0.02	0.95
C14	146.92	144.5	2.42	0.13
C15	177.97	182.5	-4.53	0.68
C17	132.52	131.1	1.42	0.80
C18	16.11	17.0	-0.89	0.94
C19	19.38	20.1	-0.72	0.85

S22

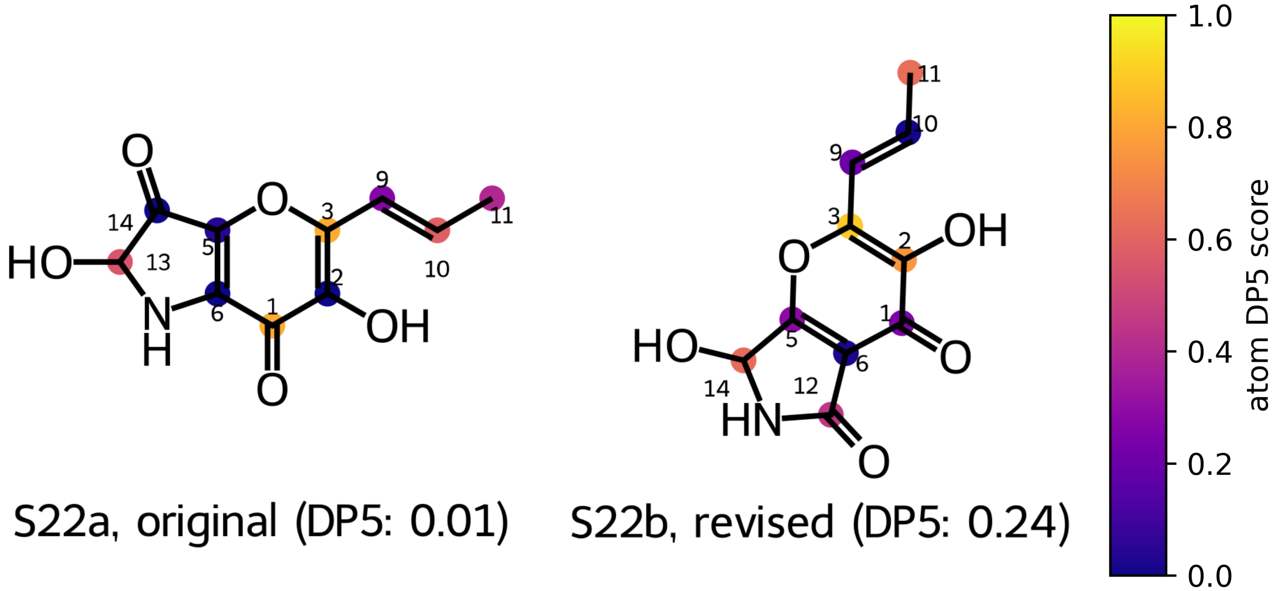

S22a, original proposal

label	calculated	assigned	error	DP5
C1	167.20	169.1	-1.90	0.82
C2	142.43	165.0	-22.57	0.00
C3	143.96	146.0	-2.04	0.82
C5	136.44	131.7	4.74	0.05
C6	127.05	111.7	15.35	0.00
C9	121.55	118.9	2.65	0.27
C10	138.98	142.2	-3.22	0.59
C11	17.06	18.7	-1.64	0.40
C13	77.08	75.2	1.88	0.55
C14	191.76	174.2	17.56	0.00

S22b, revised proposal

label	calculated	assigned	error	DP5
C1	169.57	169.1	0.47	0.26
C2	143.11	142.2	0.91	0.76
C3	144.84	146.0	-1.16	0.89
C5	178.31	174.2	4.11	0.29
C6	107.16	111.7	-4.54	0.02
C9	121.60	118.9	2.70	0.21
C10	140.18	131.7	8.48	0.01
C11	17.41	18.7	-1.29	0.64
C12	160.05	165.0	-4.95	0.45
C14	73.68	75.2	-1.52	0.63

S23

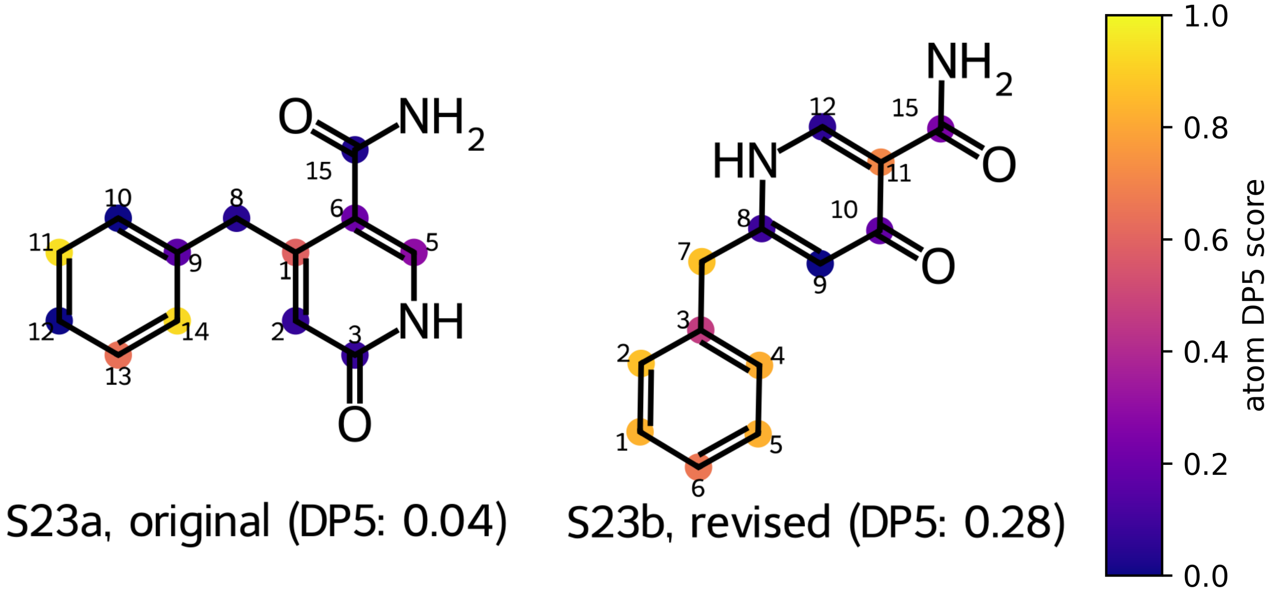

S23a, original proposal				
label	calculated	assigned	error	DP5
C1	147.35	151.2	-3.85	0.59
C2	128.27	129.3	-1.03	0.07
C3	159.82	165.9	-6.08	0.08
C5	128.80	129.2	-0.40	0.29
C6	114.84	118.0	-3.16	0.21
C8	34.53	38.2	-3.67	0.05
C9	141.15	142.2	-1.05	0.17
C10	129.29	129.3	-0.01	0.93
C11	128.31	129.2	-0.89	0.64
C12	126.41	118.9	7.51	0.00
C13	127.47	127.5	-0.03	0.94
C14	130.99	137.4	-6.41	0.00
C15	169.77	178.1	-8.33	0.03

S23b, revised proposal				
label	calculated	assigned	error	DP5
C1	129.38	129.2	0.18	0.83
C2	129.82	129.3	0.52	0.87
C3	142.62	142.2	0.42	0.46
C4	129.82	129.2	0.62	0.82
C5	129.38	129.3	0.08	0.83
C6	126.15	127.5	-1.35	0.66
C7	39.73	38.2	1.53	0.87
C8	143.88	151.2	-7.32	0.09
C9	114.02	118.9	-4.88	0.00
C10	181.07	178.1	2.97	0.19
C11	116.59	118.0	-1.41	0.71
C12	140.84	137.4	3.44	0.06
C15	160.11	165.9	-5.79	0.25

S24

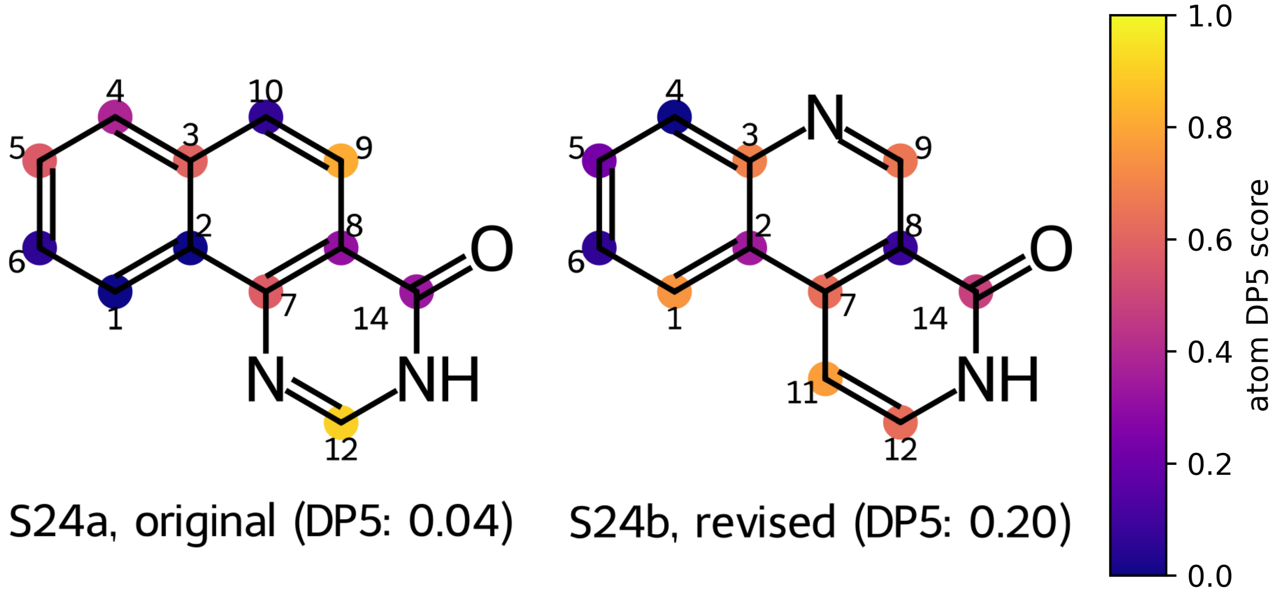

S24a, original proposal

label	calculated	assigned	error	DP5
C1	129.21	148.5	-19.29	0.00
C2	126.15	101.6	24.55	0.00
C3	131.72	136.0	-4.28	0.60
C4	127.65	128.7	-1.05	0.39
C5	130.13	130.4	-0.27	0.57
C6	127.34	125.0	2.34	0.06
C7	148.74	150.5	-1.76	0.58
C8	119.62	118.7	0.92	0.31
C9	124.80	123.6	1.20	0.82
C10	129.93	132.8	-2.87	0.07
C12	142.65	144.3	-1.65	0.90
C14	165.29	164.0	1.29	0.33

S24b, revised proposal

label	calculated	assigned	error	DP5
C1	124.34	125.0	-0.66	0.75
C2	122.23	123.6	-1.37	0.35
C3	146.35	148.5	-2.15	0.69
C4	130.42	136.0	-5.58	0.00
C5	131.34	132.8	-1.46	0.23
C6	127.66	130.4	-2.74	0.06
C7	139.13	144.3	-5.17	0.64
C8	115.75	118.7	-2.95	0.08
C9	153.09	150.5	2.59	0.66
C11	101.34	101.6	-0.26	0.77
C12	127.79	128.7	-0.91	0.63
C14	161.20	164.0	-2.80	0.48
